# Supplementary material for: Increasing sensitivity of antibody-antigen interactions using photo-cross-linking
Source: Cell Rep Methods. 2023 Jun 5;3(6):100509. doi: 10.1016/j.crmeth.2023.100509 (PMC10326447; doi:10.1016/j.crmeth.2023.100509)
Supplement: Document S1. Figures S1–S6 [file mmc1.pdf]

Cell Reports Methods, Volume *3*

Supplemental information

Increasing sensitivity of antibody-antigen interactions using photo-cross-linking

Alba Torrents de la Peña, Leigh M. Sewall, Rebeca de Paiva Froes Rocha, Abigail M. Jackson, Payal P. Pratap, Sandhya Bangaru, Christopher A. Cottrell, Subhasis Mohanty, Albert C. Shaw, and Andrew B. Ward

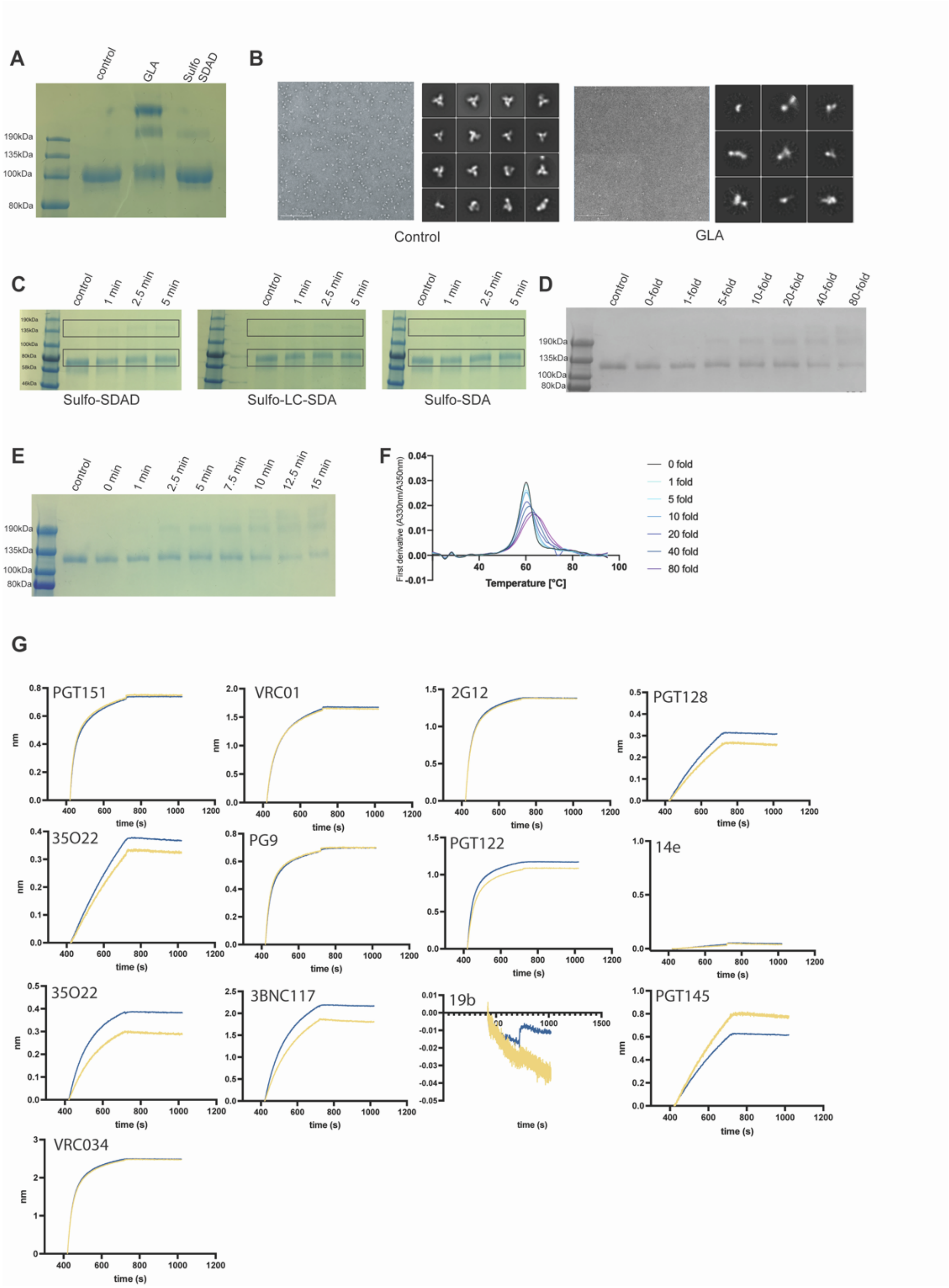

Figure S1. Screening and optimizing photo-cross-linkers using the BG505 HIV trimer. Related to Figure 1. (**A**) Non-reduced SDS-PAGE of HIV trimer using GLA and 20x fold molar excess of photo-cross-linker Sulfo-SDAD. (**B**) NS-EM micrograph and 2D classes of non-cross-linked and GLA cross-linked BG505 SOSIP.v3. (**C**) Non-reduced SDS-PAGE of HIV trimer using 20x fold molar excess of three photo-cross-linkers (Sulfo-SDAD, Sulfo-LC-SDA and Sulfo-SDA) and three UV exposure lengths: 1 min, 2.5 min and 5 min. (**D-E**) Sulfo-SDAD was used to further proceed for cross-linking. A molar range of cross-linker (panel B) and a time course UV irradiation using Sulfo-SDAD (panel C) were used to optimized cross-linking. (**F**) Nano-DSF

analysis of several cross-linker molar excess conditions was visualized by plotting the first derivative. The T_m values are listed in Fig 1. (**G**) Antigenicity was assessed by using BLI and by testing binding of a set of antibodies to the cross-linked HIV trimer or the HIV trimer alone. The antibody panel includes broadly neutralizing antibodies and non-neutralizing antibodies targeting epitopes located at different regions of the HIV trimer.

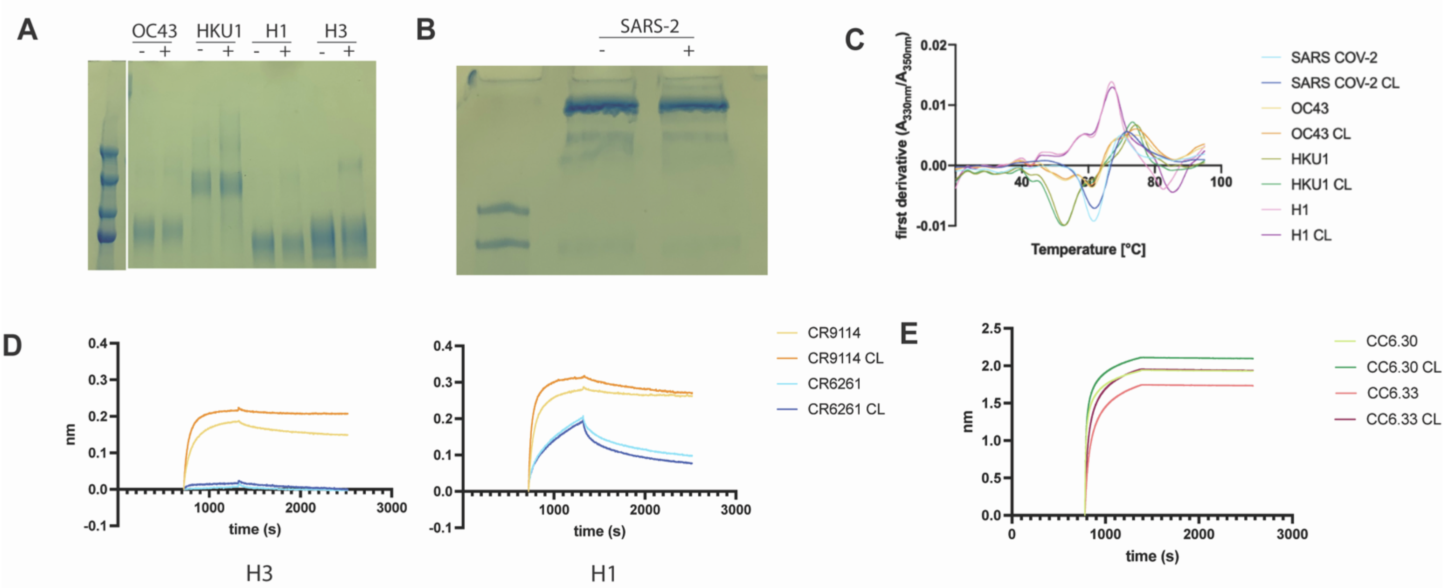

Figure S2. Biochemical and biophysical properties of cross-linked hemagglutinin and HCoV spikes.

Related to Figure 2. (**A**) Non-reduced SDS-PAGE of seasonal HCoV variants OC43 and HKU1, influenza hemagglutinin H1 and H3 and (**B**) HCoV SARS-2. (**C**) Nano-DSF analysis of HCoV and influenza HA variants is plotted. The T_m values are listed in Fig 2. (**D and E**) Antigenicity of influenza HA (D) and SARS-2 (E) was assessed by BLI using two broadly neutralizing antibodies against the HA stem (CR9114 and CR6261) and the SARS-2 RBD (CC6.30 and CC6.33), respectively.

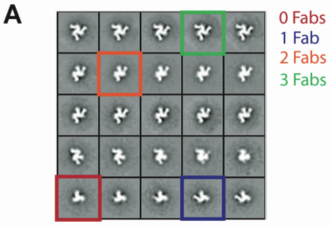

B

	Fab molar excess	number of particles	Frequency of Fab occupancy			
			0 Fab	1 Fab	2 Fab	3 Fab
CL	**0.004**	34,856	1	0	0	0
	**0.008**	40,675	1	0	0	0
	**0.016**	34,456	0.99	0.01	0	0
	**0.031**	65,195	0.96	0.04	0	0
	**0.065**	44,081	0.91	0.07	0.02	0
	**0.125**	21,411	0.83	0.15	0.01	0
	**0.25**	55,555	0.65	0.19	0.12	0.04
	**0.5**	45,221	0.43	0.29	0.22	0.06
	**1**	43,076	0.31	0.18	0.27	0.26
	**3**	50,046	0	0.01	0.3	0.69
non-CL	**0.004**	45,442	1	0	0	0
	**0.008**	39,166	1	0	0	0
	**0.016**	38,019	1	0	0	0
	**0.031**	53,407	0.98	0.02	0	0
	**0.065**	28,573	0.96	0.02	0	0
	**0.125**	25,765	0.88	0.11	0.01	0
	**0.25**	29,345	0.76	0.17	0.05	0.01
	**0.5**	52,120	0.44	0.24	0.23	0.09
	**1**	68,237	0.23	0.25	0.3	0.22
	**3**	61,186	0.03	0	0.01	0.96

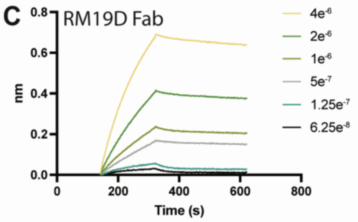

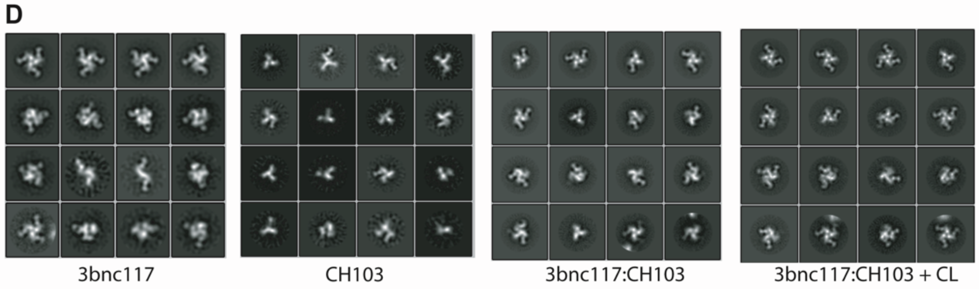

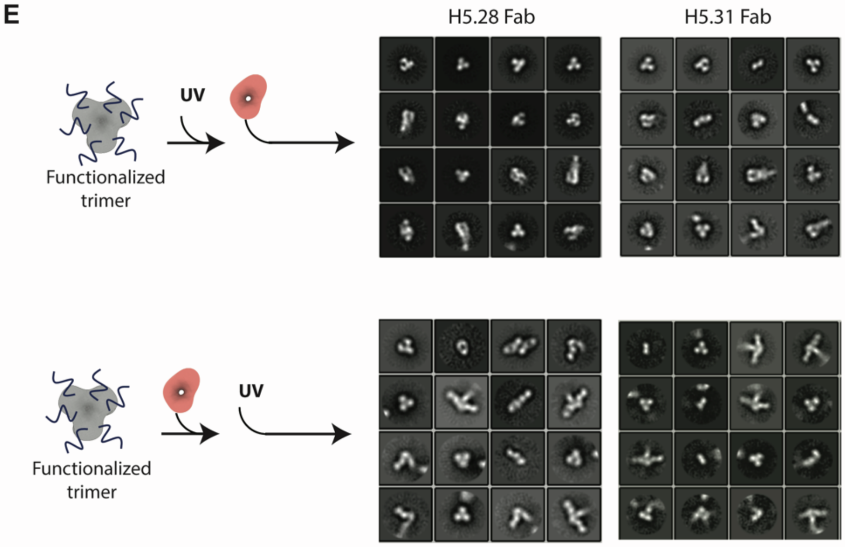

Figure S3. Biochemical and biophysical properties of the low-affinity and low-abundant Fabs. Related to Figure 3. (**A**) sample 2D class averages used to determine stoichiometry for each 3bnc117 Fab molar excess. Fab occupancy examples are highlighted. (**B**) HIV trimer occupancy distribution over a range of molar excess of 3bnc117 Fab. (**C**) Assessing affinity of the low affinity RM19D Fab by BLI. (**D**) 2D class averages of HIV trimer bound to 3bnc117 Fab and CH103 Fab (left panels). A mixture of 3bnc117 and CH103 Fabs in complex with HIV trimer in a ratio of 97:3 (3bnc117:CH103) with and without using photo-cross-linker (CL) (right panels). (**E**) 2D class averages of intermediate states of the HA protein upon H5.28 and H5.31 Fab binding.

Above, functionalization of Sulfo-SDAD to HA followed immediately by UV irradiation causes the trimer to be in a closed confirmation, with minimal antibody binding. Below, the immediate addition of two monoclonal antibodies to functionalized HA, followed UV irradiation after, allows for three antibody-antigen binding states to be visualized: 1) trimer alone, 2) HA as a dimer with two monoclonal antibodies bound and 3), HA as a monomer with a single antibody bound.

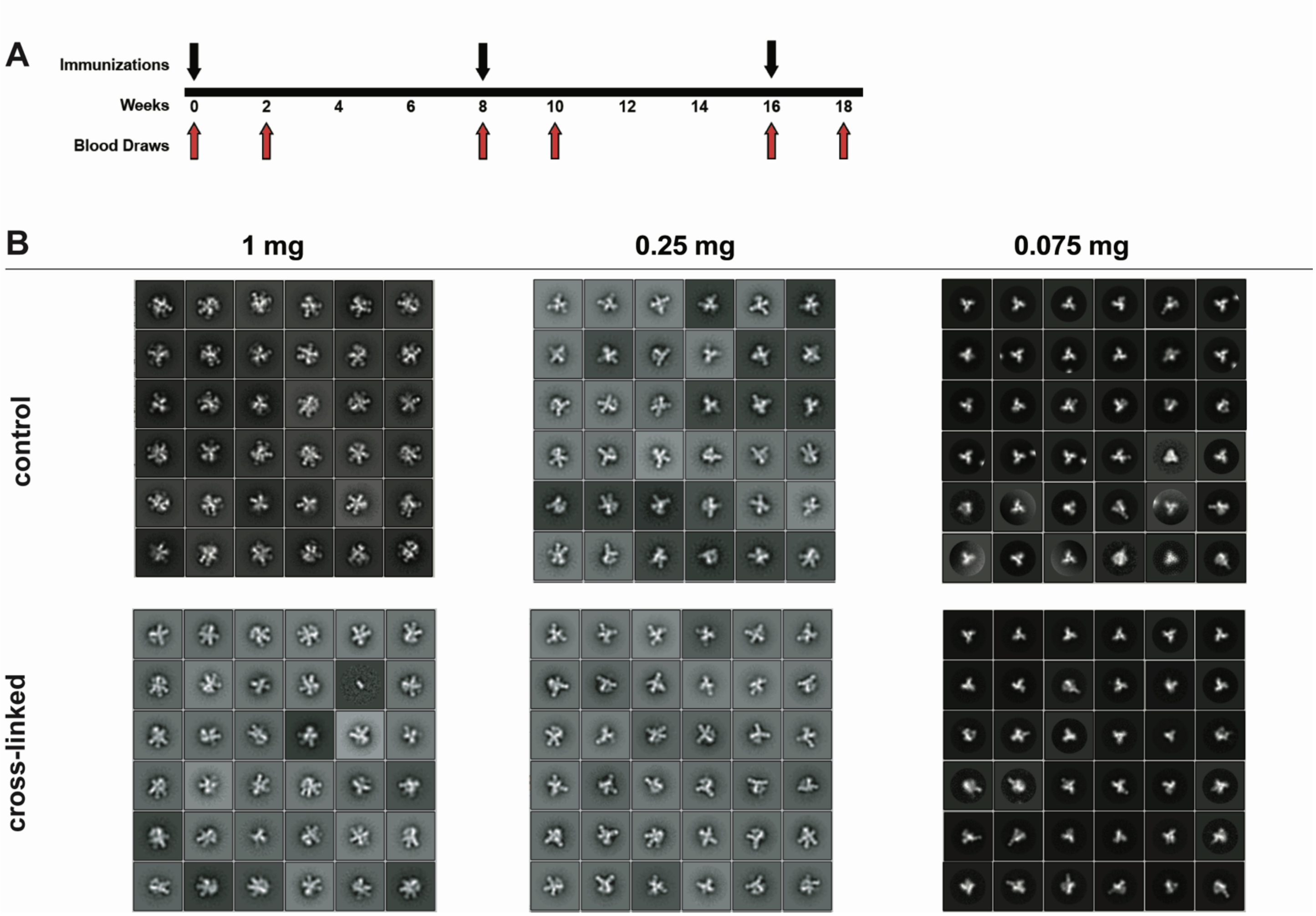

Figure S4. Immunization experiments with sera from rabbits vaccinated with the HIV immunogen. Related to Figure 4. (**A**) Immunization schedule. Red arrows represent the weeks that blood was drawn and black arrows when rabbits were immunized with the HIV trimer. (**B**) Representative 2D class averages from the NS-EM datasets used for the generation of the composite figures presented in Figure 4.

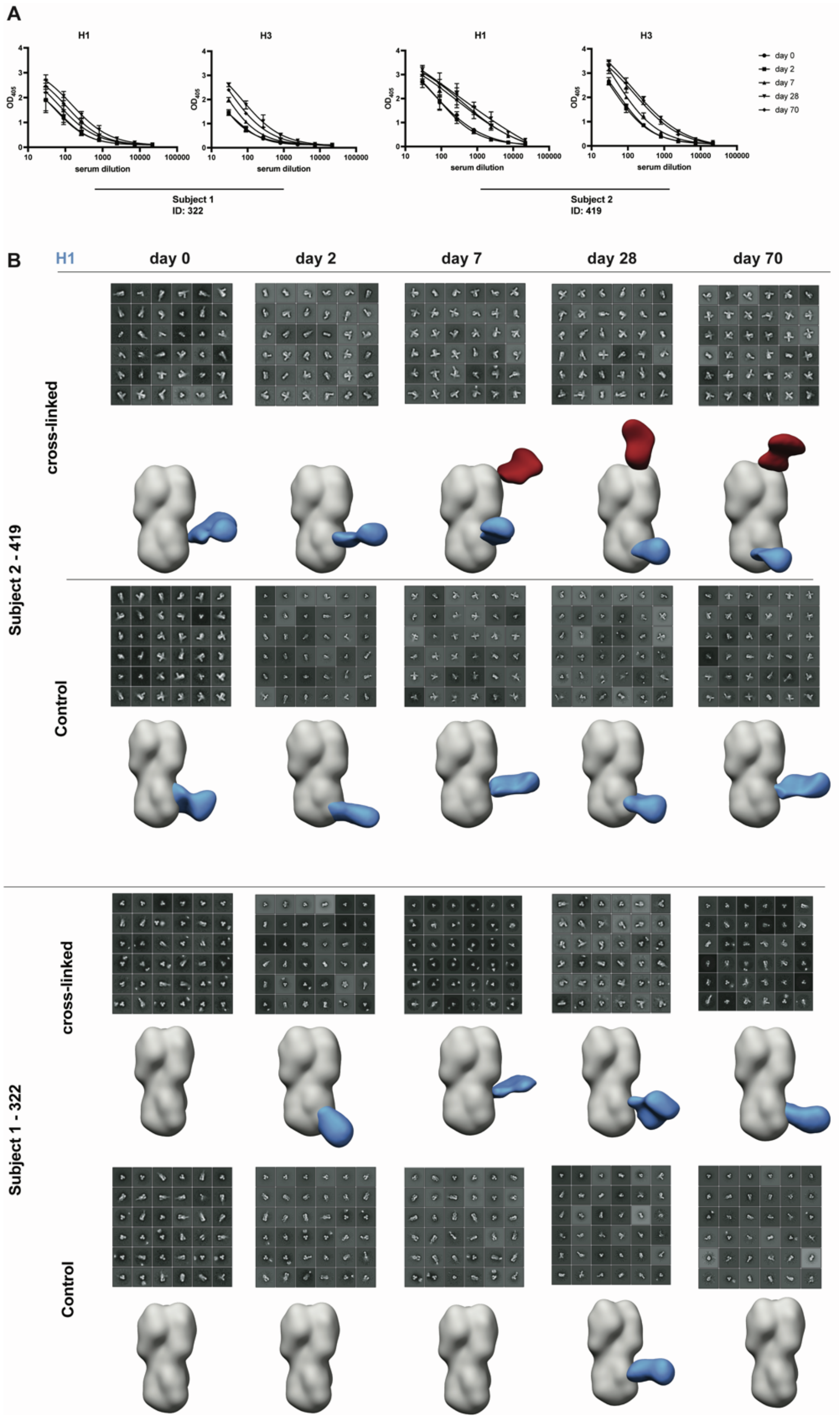

Figure S5. Extended ELISA binding titers and negative stain EMPEM analysis for HA after vaccination. Related to Figure 4. **(A)** Anti-HA binding antibody titers (EC_{50}) determined by ELISA for sera samples at different timepoints **(B-C)** Representative 2D class averages and composite models from the ns-EMPEM datasets used for the generation of the longitudinal dot plots presented in Figure 5.

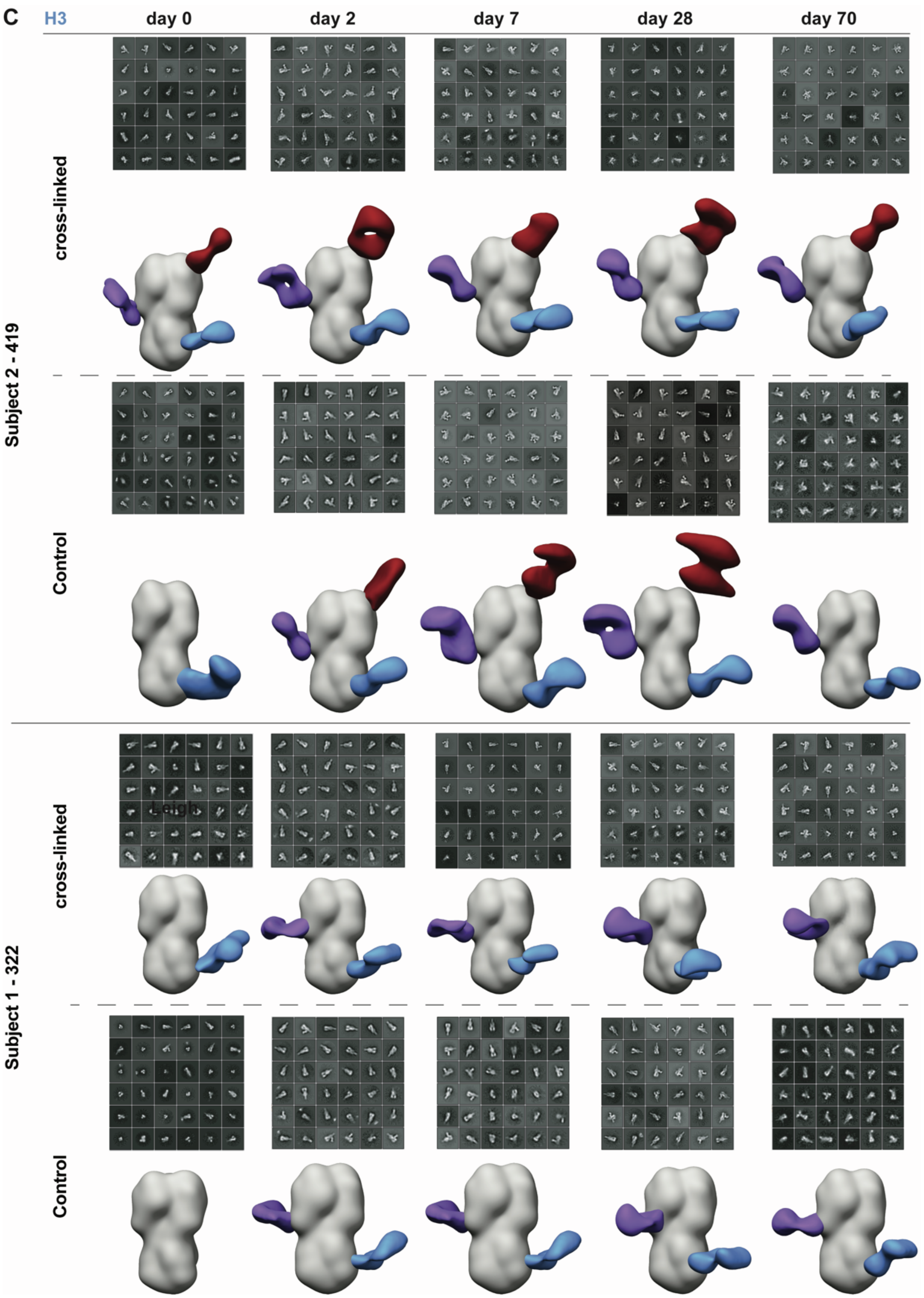

Figure S5. Extended ELISA binding titers and negative stain EMPEM analysis for HA after vaccination. Related to Figure 4. **(A)** Anti-HA binding antibody titers (EC_{50}) determined by ELISA for sera samples at different timepoints **(B-C)** Representative 2D class averages and composite models from the ns-EMPEM datasets used for the generation of the longitudinal dot plots presented in Figure 5.

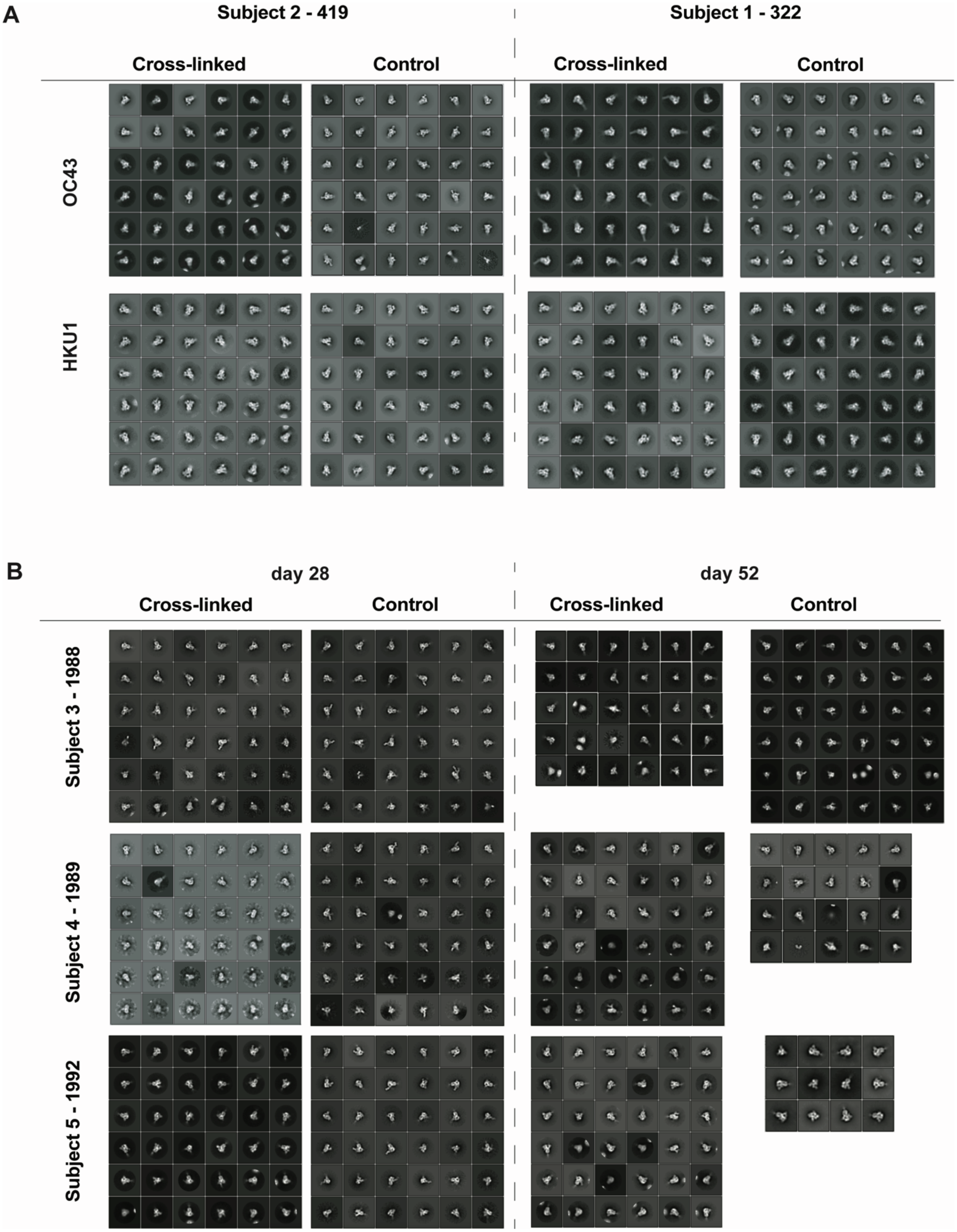

Figure S6. Extended ns-EMPEM for seasonal and pandemic HCoV during or after infection. Related to Figure 5. Representative 2D class averages from the negative stain EMPEM datasets used for the generation of composite figures of OC43, HKU1 (A) and SARS-2 (B) that are presented in Figure 5.
