# Supplementary material for: Increasing sensitivity of antibody-antigen interactions using photo-cross-linking
Source: Cell Rep Methods. 2023 Jun 5;3(6):100509. doi: 10.1016/j.crmeth.2023.100509 (PMC10326447; doi:10.1016/j.crmeth.2023.100509)
Supplement: Document S2. Article plus supplemental information [file mmc2.pdf]

Cell Reports
Methods

Article

Increasing sensitivity of antibody-antigen interactions using photo-cross-linking

Graphical abstract

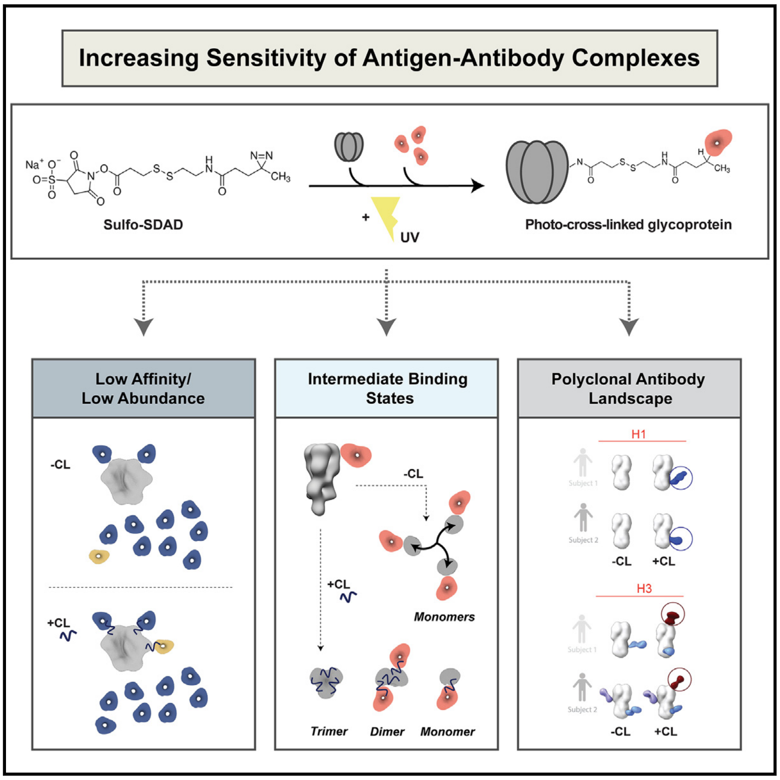

Authors

Alba Torrents de la Peña, Leigh M. Sewall, Rebeca de Paiva Froes Rocha, ..., Subhasis Mohanty, Albert C. Shaw, Andrew B. Ward

Correspondence

andrew@scripps.edu

In brief

Torrents de la Peña et al. present a method that combines electron microscopy and photo-cross-linking to stabilize antigen-antibody complexes, increasing detectability of low-abundance, low-affinity antibody specificities and detection of intermediate complex states. This technique enables more comprehensive epitope mapping of the immune response to both vaccination and infection.

Highlights

- Detection of low-abundance and low-affinity antibodies using photo-cross-linkers
- Increased detectability of antibody specificities in sera
- Stabilization of antibody-antigen complexes
- Detection of intermediate states upon antibody binding

Torrents de la Peña et al., 2023, Cell Reports Methods *3*, 100509
June 26, 2023 © 2023 The Author(s).
https://doi.org/10.1016/j.crmeth.2023.100509

Cell Reports Methods

CellPress
OPEN ACCESS

Article

Increasing sensitivity of antibody-antigen interactions using photo-cross-linking

Alba Torrents de la Peña,[1,4] Leigh M. Sewall,[1,4] Rebeca de Paiva Froes Rocha,[1] Abigail M. Jackson,[1] Payal P. Pratap,[1] Sandhya Bangaru,[1] Christopher A. Cottrell,[2] Subhasis Mohanty,[3] Albert C. Shaw,[3] and Andrew B. Ward[1,5,*]
[1]Department of Integrative Structural and Computational Biology, The Scripps Research Institute, La Jolla, CA 92037, USA
[2]Department of Immunology and Microbiology, The Scripps Research Institute, La Jolla, CA 92037, USA
[3]Department of Medicine, Section of Infectious Diseases, Yale University School of Medicine, New Haven, CT, USA
[4]These authors contributed equally
[5]Lead contact
*Correspondence: andrew@scripps.edu
https://doi.org/10.1016/j.crmeth.2023.100509

MOTIVATION Detecting low-abundance and low-affinity antigen-antibody interactions remains challenging. We present a method that combines two disciplines, single-particle electron microscopy and photo-cross-linking, to increase detectability of antibody specificities in sera after vaccination from rabbit and human serum samples. Additionally, the use of photo-cross-linking allows us to stabilize the antigen-antibody complexes and detect intermediate binding states upon antibody binding. Overall, this method enables broader detection and mapping of antibodies in serum samples after vaccination or during infection.

SUMMARY

Understanding antibody-antigen interactions in a polyclonal immune response in humans and animal models is critical for rational vaccine design. Current approaches typically characterize antibodies that are functionally relevant or highly abundant. Here, we use photo-cross-linking and single-particle electron microscopy to increase antibody detection and unveil epitopes of low-affinity and low-abundance antibodies, leading to a broader structural characterization of polyclonal immune responses. We employed this approach across three different viral glycoproteins and showed increased sensitivity of detection relative to currently used methods. Results were most noticeable in early and late time points of a polyclonal immune response. Additionally, the use of photo-cross-linking revealed intermediate antibody binding states and demonstrated a distinctive way to study antibody binding mechanisms. This technique can be used to structurally characterize the landscape of a polyclonal immune response of patients in vaccination or post-infection studies at early time points, allowing for rapid iterative design of vaccine immunogens.

INTRODUCTION

Characterizing antigen-antibody interactions is crucial to studying correlates of protection after vaccination and infection. During infection, antibodies target surface viral glycoproteins, such as influenza hemagglutinin, HIV envelope glycoprotein, or coronavirus spike protein, and can block viral entry. Therefore, defining the epitopes of these antibodies and mapping the polyclonal antibody response enables iterative structure-based vaccine design.[1] Such antibody-antigen interactions have been commonly identified using serum neutralization assays, enzyme-linked immunosorbent assays (ELISAs), and B cell sorting and electron microscopy-based polyclonal epitope mapping (EMPEM).[2–7] However, each of these techniques has limitations. First, isolation of monoclonal antibodies by B cell sorting is labor intensive and biased toward antibodies that are functionally relevant (neutralizing antibodies) or highly abundant, which results in an incomplete portrayal of the landscape of antibody responses in polyclonal sera.[2,4] Second, while EMPEM elucidates a more complex scenario of the epitopes targeted within polyclonal sera, this technique also promotes the identification of the most abundant and high-affinity antibody responses.[5–10] Thus, low-affinity, low-abundance antibodies are often lost during selection and detection.

One approach to increase detection of a protein complex involves the use of chemical cross-linking, which has been widely utilized to identify protein-protein interactions and to stabilize vaccine antigens, including the polio vaccine, influenza vaccine, diphtheria toxin, and tetanus toxin.[11–17] However, chemical cross-linking is limited because it requires specific amino acids on both

This is an open access article under the CC BY license (http://creativecommons.org/licenses/by/4.0/).

proteins at a certain distance to generate the covalent cross-links. Alternatively, the discovery of photoreactive cross-linkers such as succinimidyl-diazirine (SDA) has allowed for specific labeling of one amino acid of a targeted protein to be cross-linked to any amino acid side chain of a second non-specific target protein in close proximity.[18,19] Specifically, SDA reagents can be used to cross-link the amine-reactive N-hydroxysuccinimide (NHS) ester group of a protein with any other functional group through long-wave UV-light activation at 330–370 nm. Thus, superior specificity can be achieved with SDA photo-cross-linkers because the highly reactive intermediates allow for any amino acid side chain to cross-link to the target protein.[19]

Here, we describe a strategy to recapitulate the complexity of antigen-antibody interactions more completely, including the low-abundance and low-affinity antibodies. To achieve this, we combined the power of single-particle electron microscopy with the superior specificity of photo-cross-linking to structurally characterize monoclonal and polyclonal antibodies and generate stable antigen-antibody complexes in a serum sample. Using this technique, we show that production of these stable complexes results in increased detectability of low-affinity, low-abundance and intermediate binding states of antibodies, ultimately creating a more comprehensive and broader range map of antibody specificities in a polyclonal antibody response after vaccination or during infection.

RESULTS

Chemical and biophysical characterization of photo-cross-linked HIV envelope glycoprotein

To study photo-cross-linking induced by SDA (NHS-Diazirine [succinimidyl 4,4′-azipentanoate]) cross-linkers, we used a prototype of soluble recombinant HIV envelope (Env) glycoprotein trimers, BG505 SOSIP.v.3.[20] Recent biochemical and biophysical characterization of this soluble antigen has resulted in the discovery of many antibodies used to assess folding and antigenicity of the Env protein.[21] Therefore, this makes BG505 SOSIP.v.3 an ideal tool to test how photo-cross-linkers affect stability and antigenicity. First, we evaluated cross-linking the HIV trimer with glutaraldehyde (GLA), a methodology that has been previously described[14] and is commonly used for protein stabilization. While trimers were successfully cross-linked based on an SDS-PAGE gel, there was a portion of trimers that showed high-order aggregation (Figures S1A and S1B). Additionally, negative-stain electron microscopy indicated that most of the trimers that were cross-linked with GLA were not native-like when compared with the non-cross-linked trimers (Figure S1B). This evidence is consistent with previous GLA cross-linking studies using the HIV trimer that required an affinity purification step with a quaternary-dependent antibody to select for native-like trimers. Next, we tested three SDA photo-cross-linkers that contain three different spacer arm lengths: Sulfo-SDA (3.9 Å), Sulfo-LC-SDA (12.5 Å), and Sulfo-SDAD (13.5Å) (Figure 1A). We incubated all three photo-cross-linkers with the HIV trimer and looked for the formation of covalent bonds upon UV irradiation at 335 nm. In the absence of photo-cross-linkers, we did not detect any cross-linking (Figures 1B and S1C, lane 1). In samples irradiated with UV, we observed that Sulfo-SDAD induced the formation of substantial amounts of cross-linked dimers and trimers (23%). Conversely, fewer oligomers were formed using Sulfo-SDA and Sulfo-LC-SDA (3% and 12%, respectively; Figures 1B and S1C).

We tested a broad range of conditions with different molar excess of photo-cross-linkers and UV irradiation time and identified that 20 molar excess photo-cross-linker and 5 min UV irradiation at 1,200 μJ/s are optimal for maximizing the efficiency of covalent bonds while avoiding the formation of non-specific aggregates (Figures 1D–1F and S1D–S1F). Using the optimized protocol, quantification of free amines upon UV irradiation showed that ∼60% were modified and linked to the photo-cross-linked HIV trimer (Figure 1C). Since the trimer contains a total of 201 lysine and arginine amino acids, approximately 120 amino acids were therefore cross-linked using this protocol (Figure 1C). The photo-cross-linking process resulted in a more heat-resistant HIV trimer with a melting temperature increased by 3.5°C compared with the non-cross-linked counterpart (non-cross-linked, 66.7°C; cross-linked, 70.2°C) (Figures 1E and S1F), consistent with intramolecular cross-links within the HIV trimer.

Next, using a panel of monoclonal antibodies that included broadly neutralizing and non-neutralizing antibodies, we compared the antigenicity of the BG505 SOSIP.v.3 cross-linked and non-crosslinked trimers by biolayer interferometry (BLI). While broadly neutralizing antibodies (bNAbs) (2G12, PGT122, PGT128, 3BNC117, VRC01, 35O22, PG9, PGT145, PGT151, and VRC034) bound to the HIV trimer, non-neutralizing antibodies (14e and 19b) targeting inaccessible epitopes in a well-folded trimer did not bind to either the photo-cross-linked (Sulfo-SDAD) or non-cross-linked HIV trimer. Importantly, although most of the antibodies bound similarly to the cross-linked trimer when compared with its non-cross-linked counterpart, the bNAb PGT145 showed less binding to the cross-linked trimer, likely due to modification of one or more lysines within the PGT145 epitope (Figures 1G and S1G).

Extending photo-cross-linking to stabilize additional viral glycoproteins

Next, we explored the efficiency of photo-cross-linkers with additional viral glycoproteins to evaluate the effect photo-cross-linkers have on the structure and antigenicity of these glycoproteins. Thus, we examined the efficiency of photo-cross-linkers with human coronavirus (HCoV) spike proteins and influenza hemagglutinins (HAs). We began by using the previously optimized method for the HIV trimer. Comparable to the HIV trimer, HCoV spikes OC43, HKU1, and severe acute respiratory syndrome coronavirus 2 (SARS-CoV-2) showed efficient photo-cross-linking with minimal aggregation (Figures 2A and S2A). However, HA trimers from two groups, H1 and H3, degraded into monomers upon UV irradiation (Figure S2A). To further investigate this, we treated the trimeric HAs with different UV irradiation power and characterized their integrity. Biophysical and biochemical properties of HA and HCoV trimers were further assessed via the free amine assay, negative-stain electron microscopy (NS-EM), nano-differential scanning fluorimetry (DSF), and BLI. First, all viral antigens showed binding of the photo-cross-linker to their amine groups (Figure 2B). Second, analysis of 2D classification using NS-EM showed that all trimers were native-like, indicating that photo-cross-linking did not

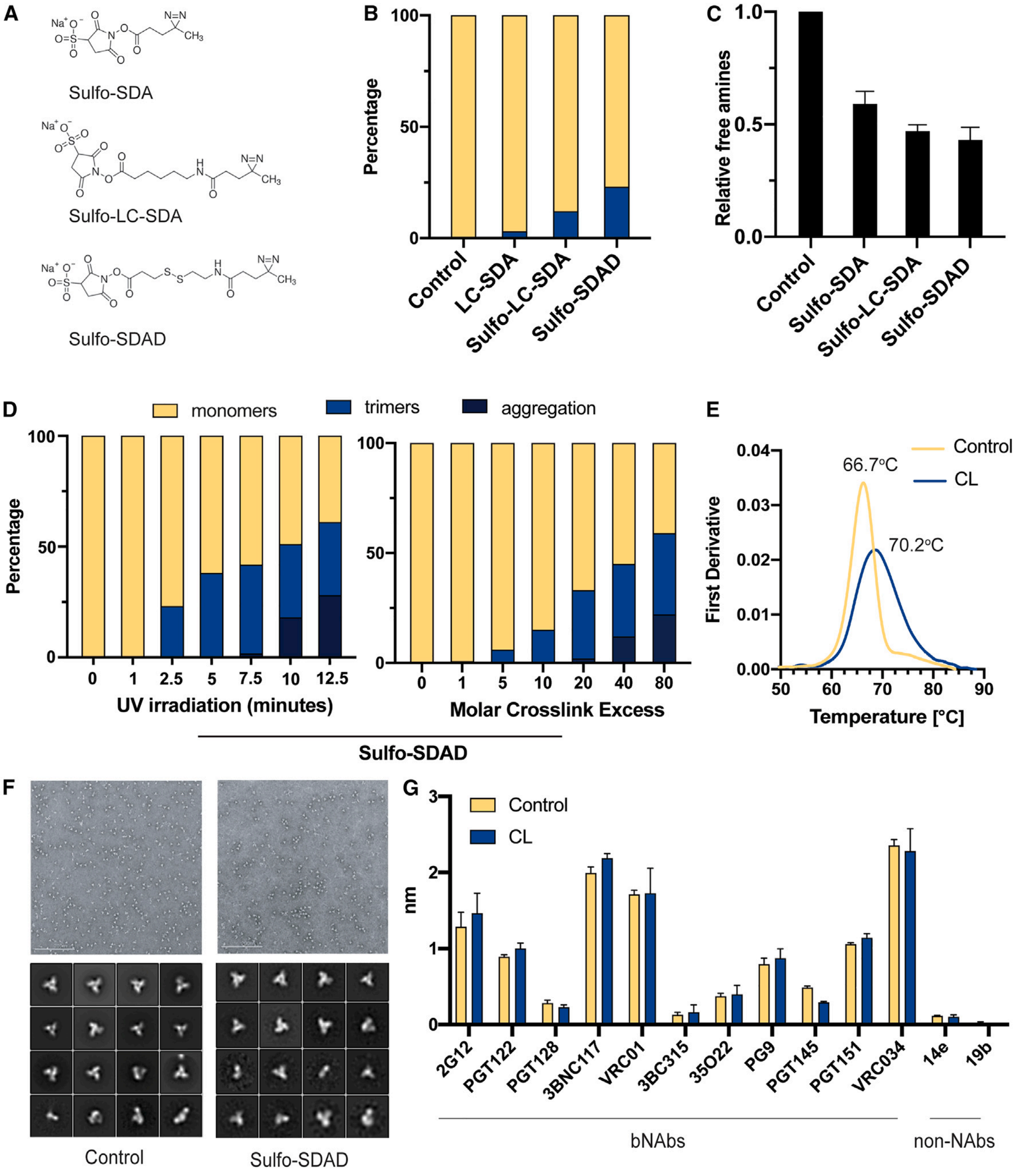

Figure 1. Stabilization of HIV trimers using photo-cross-linkers

(A) Chemical structure of three SDA photo-cross-linkers tested for use in photo-cross-linking technique. Sulfo-SDA includes a 3.5 Å arm length, Sulfo-LC-SDA a 12.5 Å arm length, and Sulfo-SDAD a 13.5 Å arm length.

(B) Percentage of cross-linked HIV BG505 SOSIP.v.3 trimers that oligomerized upon UV irradiation (335 nm) in the presence of each photo-cross-linker. Experiments were performed in triplicate. Data are represented as mean ± SEM.

(C) Percentage of relative free amines on the surface of BG505 SOSIP.v.3 with the addition of the three cross-linkers; a lower amount of relative free amines indicates an increase in cross-linked amines on the trimer. Experiments were performed in triplicate. Data are represented as mean ± SEM.

(D) Time course of UV irradiation using Sulfo-SDAD functionalized BG505 SOSIP.v.3 (left panel) and molar range of cross-linker in relation to the trimer (right panel).

(E) Relative melting temperature of BG505 SOSIP.v.3 trimer when Sulfo-SDAD is cross-linked to the trimer using nano-DSF.

(F) NS-EM micrograph and 2D classes of cross-linked and non-cross-linked BG505 SOSIP.v.3.

(G) A panel of monoclonal antibodies was used to demonstrate binding capabilities of bNAbs and non-NAbs to the BG505 SOSIP.v.3 by BLI. Experiments were performed in duplicate. Data are represented as mean ± SEM.

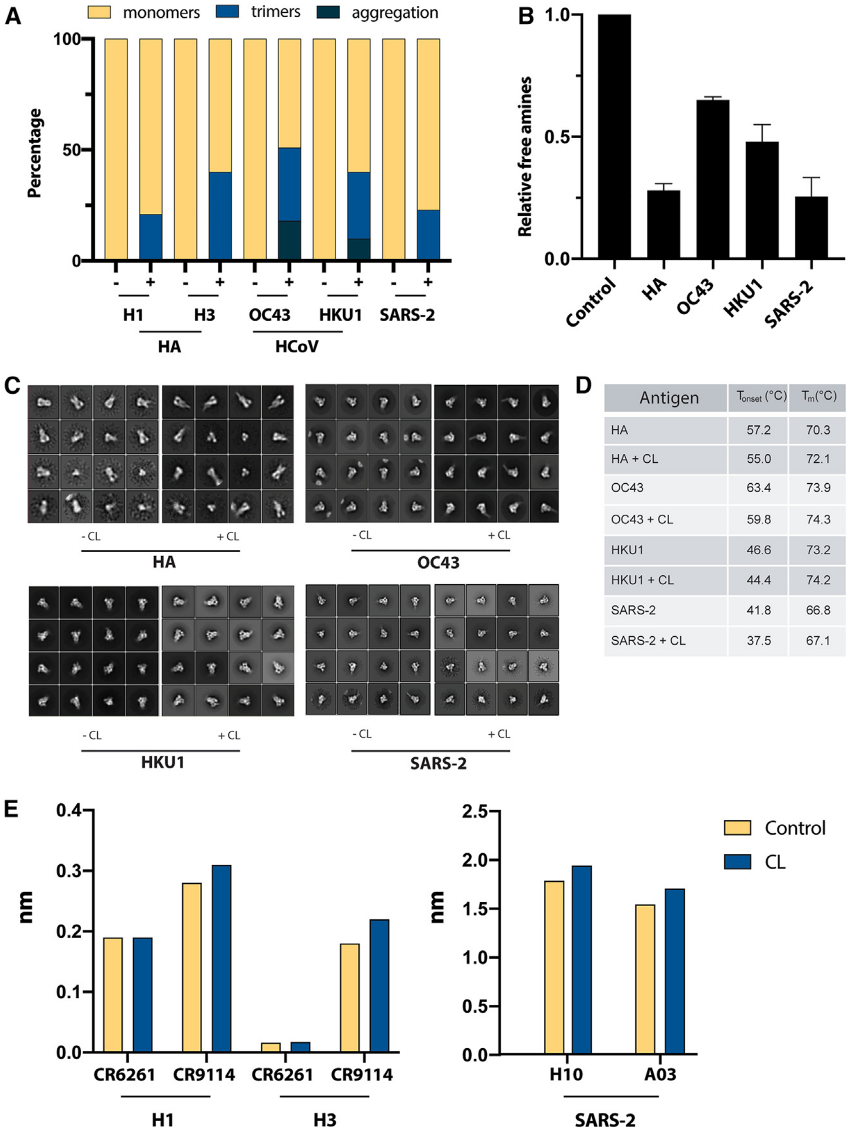

Antigen	T_{onset} (°C)	T_m(°C)
HA	57.2	70.3
HA + CL	55.0	72.1
OC43	63.4	73.9
OC43 + CL	59.8	74.3
HKU1	46.6	73.2
HKU1 + CL	44.4	74.2
SARS-2	41.8	66.8
SARS-2 + CL	37.5	67.1

Figure 2. Stabilization of various viral glycoproteins using photo-cross-linkers

(A) Percentage of cross-linked glycoproteins—H1 and H3 influenza hemagglutinins (HAs) and spike proteins from human betacoronavirus OC43, HKU1, and SARS-2—that oligomerized upon UV irradiation (335 nm) in the presence of all three cross-linkers. Experiments were performed in triplicate. Data are represented as mean ± SEM.

(B) Percentage of relative free amines on the surface of HA, OC43, HKU1, and SARS-2 with the addition of the Sulfo-SDAD cross-linker; a low percentage of relative free amines indicates an increase in cross-linked amines on the trimer.

(C) Representative NS-EM micrographs and 2D classes of photo-cross-linked and non-photo-cross-linked glycoproteins.

(D) Relative thermal stability of HA, OC43, HKU1, and SARS-2 glycoproteins increases when Sulfo-SDAD is cross-linked to the trimer, as observed by an increase in melting temperatures via nano-DSF.

(E) Using BLI, two monoclonal antibodies were used to demonstrate binding capabilities to each H1 and H3 strain of HAs and SARS-2 spike protein, respectively, indicating the trimeric glycoproteins remained unchanged in the presence of a Sulfo-SDAD cross-linker.

compromise the structural integrity of trimers (Figure 2C). Third, trimer thermostability increased upon photo-cross-linking, especially in HA trimers. While HKU1, OC43, and SARS-2 showed an increase of T_m values of 1°C, 0.4°C, and 0.3°C, respectively, photo-cross-linking of HA improved trimer thermostability by 1.8°C compared with its counterparts (Figures 2D and S2C). Finally, antigenicity of HA and HCoV spikes using nAbs showed similar profiles for photo-cross-linked and non-cross-linked trimers (Figures 2E, S2D, and S2E). Taken together, using the SDAD photo-cross-linker improved stability of the additional glycoprotein antigens and did not appear to occlude antibody-binding ability.

Photo-cross-linking increases detection of low-affinity antibodies

To evaluate whether antibody detection was impacted by photo-cross-linking, we assessed binding of HIV Env trimers with different molar ratios of the high-affinity monoclonal antibody 3BNC117 in the presence or absence of a photo-cross-linker. 3BNC117 targets the CD4 receptor-binding site on the HIV Env glycoprotein, and because HIV trimers contain three receptor-binding sites, three 3BNC117 Fabs (fragments antigen binding) can potentially bind a single trimer. 3BNC117 Fab levels were assessed by NS-EM and quantified as previously described.[22] Overall, photo-cross-linking the Fab to the viral antigen enabled Fab detection at similar molar ratios compared with non-cross-linked complexes (Figures 3A, 3B, S3A, and S3B). Hence, the presence of the photo-cross-linker on the HIV trimer did not interfere with antibody binding despite the potential modification of lysines within antibody epitopes. Moreover, we assessed whether photo-cross-linking allows visualization of low-abundance, low-affinity antibodies in a mixture with high-affinity and high-abundance antibodies. To test this, we used a mixture of the high-affinity antibody 3bnc117 Fab (EC_{50} of 102 ng/mL; Sanders et al.[20]) and the low-affinity antibody CH103 Fab (EC_{50} of 742 ng/mL, Sanders et al.[20]). While binding of antibodies was similar between the cross-linked and non-cross-linked samples in a mixture of 95:5 (3bnc117:CH103), CH103 Fab was only detectable in the cross-linked sample in a mixture of 97:3 (3bnc117:CH103) (Figure S3D). Thus, photo-cross-linking can detect low-abundance and low-affinity antibodies in a mixture.

Next, to investigate detection of low-affinity interactions, we used the SDA photo-cross-linker to covalently link the HIV trimer

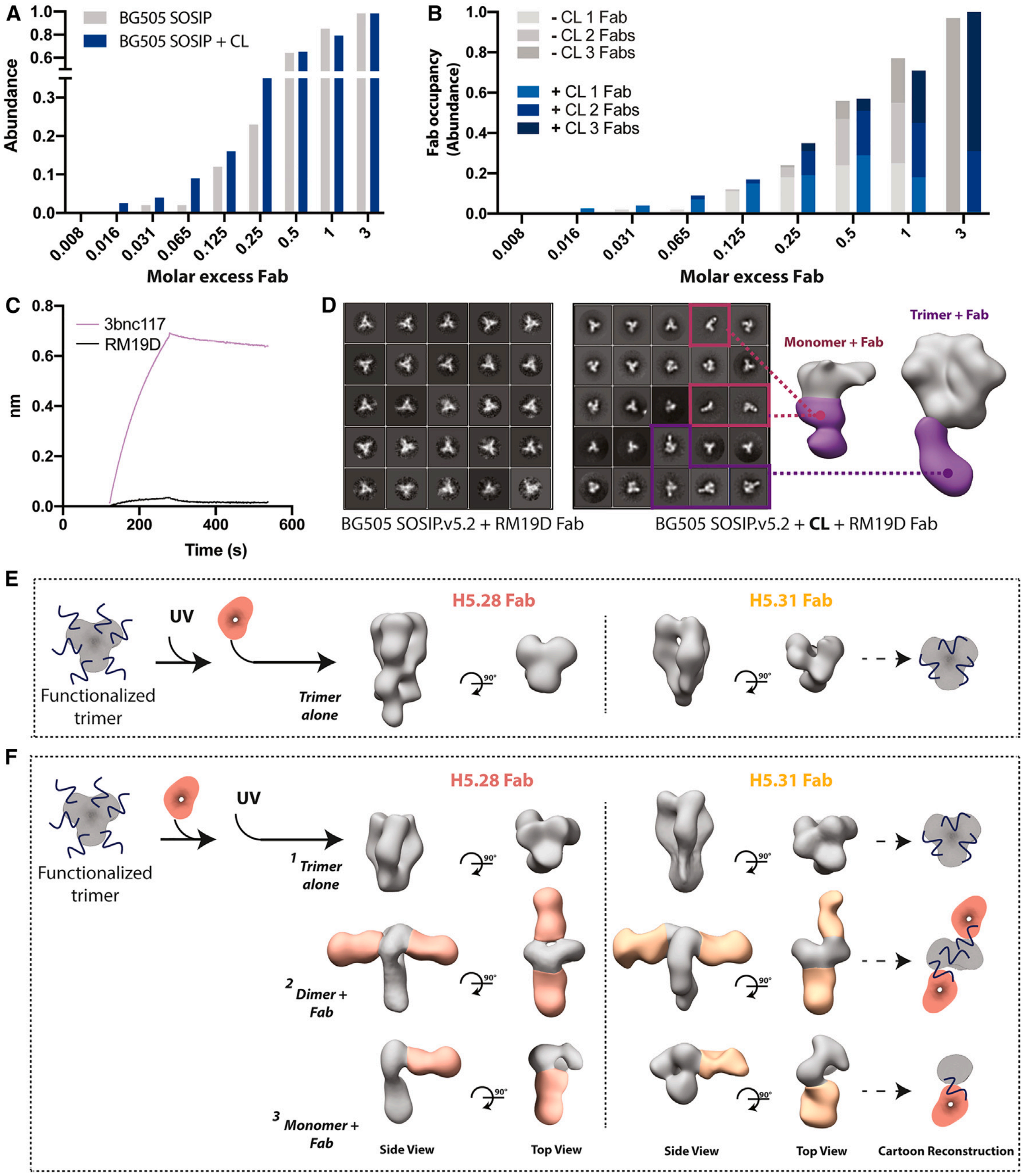

Figure 3. Limit of detection of photo-cross-linked antibody-antigen complexes

(A) Limit of detection was determined by decreasing the molar excess of a high-affinity monoclonal antibody using Sulfo-SDAD cross-linked and non-cross-linked BG505 SOSIP.v.3, reported as a frequency of abundance.

(B) Monoclonal antibody occupancy was determined by decreasing the molar excess of the same monoclonal antibody using cross-linked and non-cross-linked BG505 SOSIP.v.3. A maximum of three antibodies can be bound to one trimer at any molar excess.

(C) Using BLI, the K_D of two monoclonal antibodies with high (3BNC117 Fab) and low (RM19D Fab) affinities was determined.

(D) 2D classification and 3D reconstruction via NS-EM of the low-affinity RM19D monoclonal antibody.

(E) Functionalization of Sulfo-SDAD to HA followed immediately by UV irradiation causes the trimer to be in a closed confirmation, with no antibody binding visible.

(F) The immediate addition of two monoclonal antibodies to functionalized HA, followed by UV irradiation after, allows for two antibody-antigen binding states to be visualized: (1) trimer alone, (2) HA as a dimer with two monoclonal antibodies bound, and (3) HA as a monomer with a single antibody bound.

to a low-affinity monoclonal antibody, RM19D immunoglobulin G (IgG; K_D: 1.6×10^{-7})[9] (Figures 3C and S3C), the epitope of which has remained unknown[9] (Figure 3C). Here, however, photo-cross-linking to BG505 SOSIP.v.5.2 enabled mapping the epitope of RM19D (Figure 3D), revealing that RM19D targets the base of the HIV trimer (Figure 3D). Moreover, we observed a subset of particles that were monomer subunits of the BG505 SOSIP trimer bound to RM19D Fabs, suggesting that the binding mechanism of this Fab induces the HIV trimer to fall apart into monomers (Figure 3D), consistent with recent observations of base-directed antibodies.[23] Both observations were only possible when using the photo-cross-linked version of the HIV trimer.

Identification of conformational changes in influenza HA trimer upon antibody binding

Photo-cross-linking technology can also be used to study conformational changes upon antibody interaction with an antigen. Previous studies demonstrated that specific types of monoclonal antibodies target the trimer interface epitope of influenza HA, which results in rapid dissociation of the trimers into Fab-bound monomeric HA subunits.[24–26] Due to this rapid transition from trimeric to monomeric states, intermediate states of the induced antibody-antigen conformational changes have remained elusive. Here, we used photo-cross-linking to investigate the conformational changes of HA derived from strain H1 A/California/7/2009 bound to two different broadly reactive monoclonal antibodies, H5.28 Fab and H5.31 Fab, both isolated from patients vaccinated with the A/Vietnam/1203/2004 H5N1 (VN/04) subunit vaccine.[24] Since the Cal09 H1 trimer falls apart upon binding to H5.28 and H5.31 Fabs,[26] we functionalized the Cal09 H1 trimer, exposed it to UV irradiation at 600 μJ/min for 5 min, and complexed it with H5.28 or H5.31 Fabs. EM images revealed only apo Cal09 H1 trimers, indicating that the trimer interface epitope is inaccessible in photo-cross-linked, closed HA trimers (Figures 3E and S3E). Next, we functionalized the Cal09 H1 trimer, complexed it with H5.28 or H5.31 Fabs, and immediately exposed the complex to UV irradiation at 600 μJ/min for 5 min. Using this process, we visualized three different species of antibody binding to the antigen for both monoclonal antibodies: an apo HA trimer, a dimer bound to two Fabs, and a Fab-bound monomeric subunit (Figures 3F and S3E). Together, these findings suggest that during transient breathing of the HA trimer, the trimer interface becomes accessible. This is in line with previous studies demonstrating that the HA head can breathe or open up and reveal hidden epitopes in the HA head domain that are targeted by broadly protective antibodies.[25,27,28] Antibody binding to one HA head domain results in the dissociation of one protomer, allowing two antibodies to bind the dimer. This binding event results in subsequent dissociation into multiple Fab-bound monomeric subunits. Importantly, the intermediate Fab-bound dimeric states were only visualized when the HA trimer was functionalized with the SDAD photo-cross-linker.

Mapping the landscape of epitopes in a polyclonal immune response after vaccination or infection

Given the results with monoclonal antibodies, we next tested whether photo-cross-linker technology could be applied to increase the sensitivity of antigen-antibody interactions in polyclonal sera. First, we evaluated sera from a rabbit that was primed and boosted with HIV trimer at weeks 0, 8, and 16 (Figure S4). We complexed the HIV trimer with decreasing amounts of polyclonal sera that were collected at week 18 to determine how photo-cross-linking impacted detection of epitopes in a polyclonal immune response. Following incubation of HIV trimers with 1 mg of polyclonal Fabs, we identified six epitopes: base, interface (A316W-stabilizing mutation), N611 glycan hole, fusion peptide, N241/N289 glycan hole, and CD4-binding site (Figures 4A and S4B). We then photo-cross-linked the HIV trimer with 1 mg of polyclonal Fabs for 1 min or 24 h after complexing and detected the same six epitopes. However, when we photo-cross-linked the HIV trimer with 1 mg of polyclonal Fab following a 24 h incubation, we had to acquire more particles (200,000 particles vs. 100,000 particles) to detect the CD4-binding site epitope. This could be a result of a greater abundance of interface antibodies in the serum or relatively higher affinity and superior binding kinetics of interface antibodies. Next, we complexed 0.25 mg polyclonal Fabs with the HIV trimer and identified three of the six epitopes present previously, including base, N611 glycan, and fusion peptide responses. Identical epitopes were present with and without a photo-cross-linker (Figures 4A and S4B). Finally, when the HIV trimer was incubated with 0.075 mg of polyclonal Fabs and photo-cross-linked, the same three epitopes were detected (Figures 4A and S4B). However, only one epitope, base, was detected when the HIV trimer was complexed without using the photo-cross-linker (Figures 4A and S4B), indicating that the use of photo-cross-linking technology can improve the detection of low-abundance or highly diluted antibodies in polyclonal sera.

Next, we obtained sera from two human subjects that participated in a seasonal flu vaccination study (subject 1: ID 322, subject 2: ID 419), where a high-dose trivalent flu vaccine (Fluzone) was administered at day 0 and sera were collected at days 0, 2, 7, 28, and 70 (Figure 4B). We initially assessed the polyclonal immune response by performing traditional serological analyses and structurally characterizing the epitopes targeted over time. First, we observed that antibody titers (IgG) increased until day 28, followed by a decrease through day 70 (Figures 4C and S5A). HA inhibition (HAI) titers showed a comparable pattern, with values peaking at day 28 and decreasing afterward (Figure 4D). Next, to reveal the epitope specificities as well as the dynamics of the polyclonal antibody response over time, we used EMPEM without the SDAD photo-cross-linker.[8] We studied two subjects, subject 1 (ID 322) and subject 2 (ID 419), and complexed the isolated polyclonal antibodies (pAbs) with HA from matching vaccine strains: H1 (A/Michigan/045/15) and H3 (A/INFIMH/16). For subject 1, we only observed an H1-specific stem antibody response at day 28, which disappeared by day 70 (Figures 4E and S5B). Additionally for subject 1, we detected H3 stem- and esterase-specific antibody responses from day 2 onward. Subject 2 showed a stem antibody response against H1 and H3 at day 0, which persisted until day 70 (Figures 4E and S5B). Head antibody responses to H1 emerged at day 28, and head and esterase antibody responses were observed at day 28 until day 70 for H3

CellPress
OPEN ACCESS

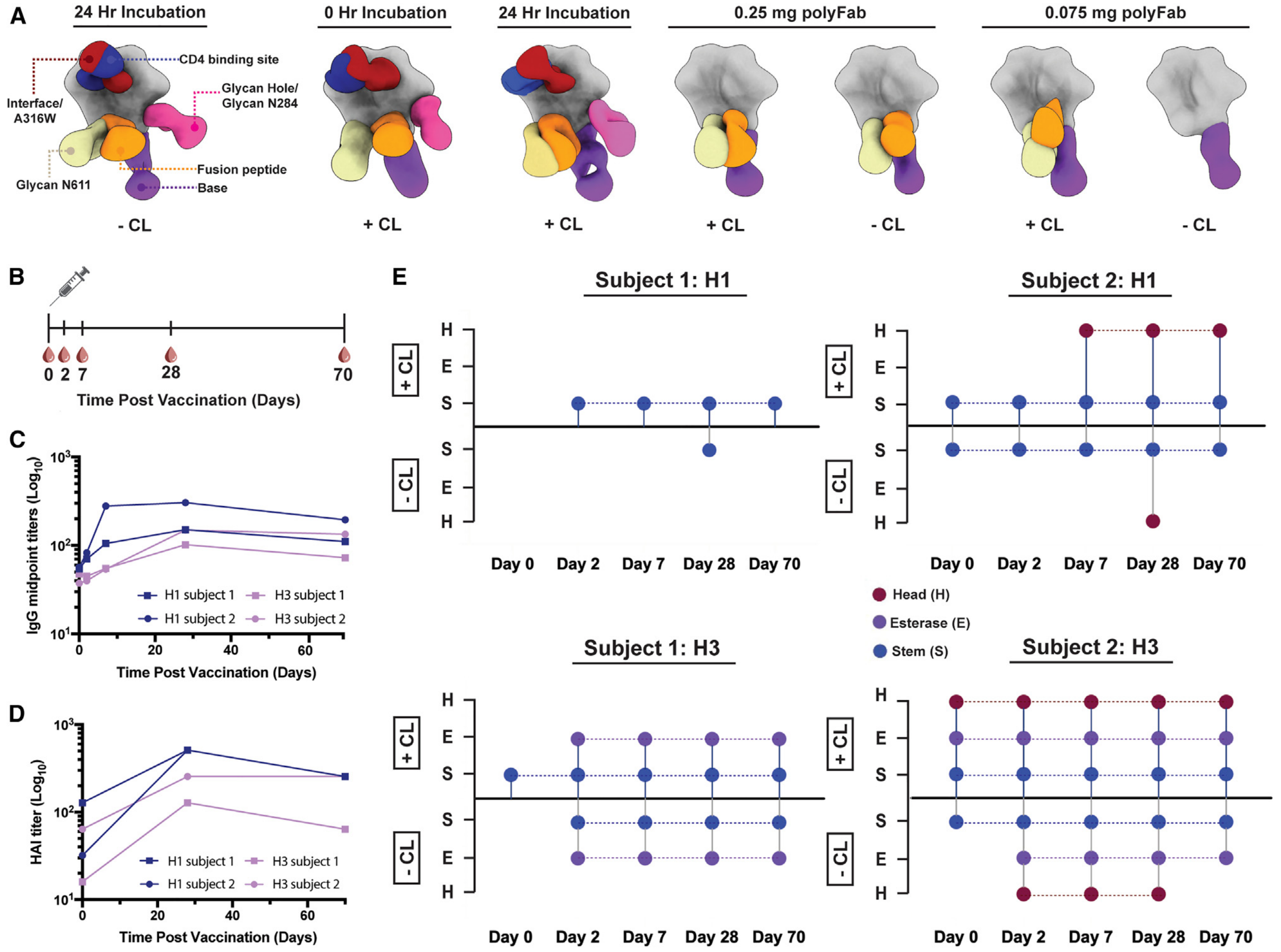

Figure 4. Detection of epitopes targeted in a polyclonal antibody response against HIV and HA after vaccination

(A) Analysis of polyclonal immune responses against the HIV trimer 2 weeks after vaccination with BG505 SOSIP. Composite figures from NS EMPEM analysis of polyclonal responses when the HIV trimer was incubated with 1, 0.25 mg, or 0.075 mg polyclonal sera immediately or for 24 h. Composite figures are shown for samples that were complexed with (+CL) or without (−CL) a photo-cross-linker. A color-coding scheme for antibodies targeting different epitope clusters is shown in the left panel. The HIV trimer is represented in gray.

(B) Vaccination timeline with injection at day 0 and blood draws at days 0, 2, 7, 28, and 70.

(C) IgG midpoint titers over time determined via ELISA.

(D) HAI titers over time.

(E) Longitudinal dot plots of EMPEM responses observed in two subjects. HA that was cross-linked via Sulfo-SDAD functionalization is presented in the positive y axis direction and labeled "+CL". HA that was not cross-linked is shown in the negative y axis direction and labeled "−CL". Labels presented on the y axis correspond to epitopes indicated in the figure legend (D). Antibody responses were assessed between days 0 and 70 and against HA strains H1 and H3.

(Figures 4E and S5B). Overall, the dynamics of the pAb responses mapped by NS-EM were consistent with the ELISA and HAI data, which showed that antibodies increased after vaccination and waned after day 28.

Serological analyses indicated that antibodies were present against H1 and H3 at day 0 in both patients, but we could not visualize the epitopes targeted by these antibodies using the traditional EMPEM approach. Thus, we employed the use of photo-cross-linking to probe for low-abundance HA-specific antibodies most likely present in the polyclonal immune response in these same two patients. First, we functionalized vaccine matching strains of H1 and H3 with the SDAD photo-cross-linker, complexed these viral antigens with polyclonal sera from subjects 1 and 2, and performed UV irradiation. For both subjects, we detected additional antibody specificities in early and late time points post-vaccination (Figures 4E, S5B, and S5C). For subject 1, we detected a stem antibody against H1 at day 2 and against H3 at day 0 (compared with days 28 and 2 for non-cross-linked complexes, respectively) (Figures 4E, S5B, and S5C). For subject 2, we detected an additional antibody specificity against the head of H1 and against the stem and esterase of H3 at days 7 and 0, respectively (Figures 4E, S5B, and S5C). Notably, the use of a photo-cross-linker to detect antibodies targeting H1 and H3 pre-vaccination to day 7, many of which are not observed without a cross-linker, provides the possibility that this technique may be used to study pre-existing immunity or immune

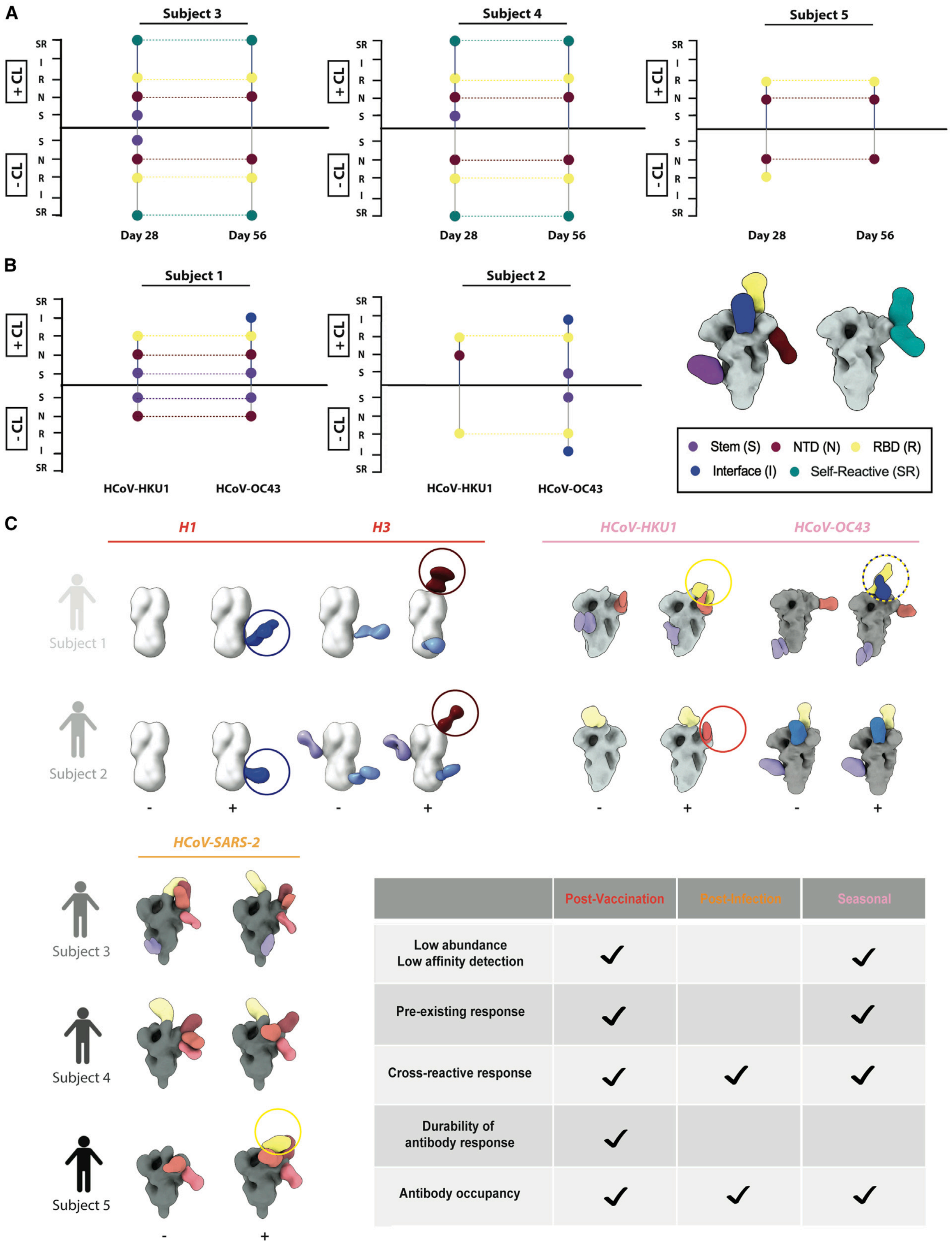

	Post-Vaccination	Post-Infection	Seasonal
Low abundance Low affinity detection	✓		✓
Pre-existing response	✓		✓
Cross-reactive response	✓	✓	✓
Durability of antibody response	✓		
Antibody occupancy	✓	✓	✓

(legend on next page)

imprinting. Taken together, these results demonstrate that the use of photo-cross-linkers to complex viral antigens with polyclonal antibodies (pAbs) increases sensitivity of epitope detection in serum.

To further expand the photo-cross-linking technology, we next used three different functionalized HCoV spike proteins to probe for seasonal antibody responses in patient sera. We obtained sera from the same two human subjects that participated in the previous seasonal flu vaccination study, subjects 1 and 2 (IDs 322 and 419, respectively), as well as from three convalescent donors, subjects 3, 4, and 5 (IDs 1988, 1989, and 1992, respectively) who had been infected with HCoV-SARS-2.[5,29] We used two human betacoronavirus spike proteins, HCoV-HKU1 and HCoV-OC43, that seasonally circulate in the general population, as well as the human betacoronavirus spike protein HCoV-SARS-2, responsible for the ongoing COVID-19 pandemic. Upon HCoV-SARS-2 infection, antibody titers (IgG) increased until 28 and remained elevated through day 56.[5] Structural analysis via EMPEM showed no differences in antibody detection with or without a photo-cross-linker in subjects 3 and 4 at either day 28 or 56 when complexed with SARS-2 (Figures 5A–5C and S6B). In subject 5, N-terminal domain (NTD)- and receptor-binding domain (RBD)-targeting antibodies were observed at day 28 in both conditions; however, at day 56, the RBD specificity to the SARS-2 spike protein was only detected in the presence of the photo-cross-linker (Figures 5A–5C and S6B).

Subsequently, we analyzed whether antibodies derived from a seasonal infection with OC43 or HKU1 could be detected with or without the presence of the SDA photo-cross-linker. Using sera from subjects 1 and 2 at day 0 post-vaccination, we observed pre-existing antibodies against HCoV strains. For both subjects 1 and 2, when HKU1 was photo-cross-linked with pAbs, we detected an additional epitope (Figures 5A and S6A). Additionally, for subject 1, two additional epitope specificities were detected when we cross-linked OC43 with the pAbs compared with the non-cross-linked counterpart (Figures 5B and S6A). The increased sensitivity of epitope detection at early and late post-vaccination time points helps structurally inform three biologically relevant settings: pre-existing immunity, the effectiveness of vaccine priming, and the durability of the immune response.

DISCUSSION

Here, we have introduced the use of photo-cross-linkers as a strategy to improve the sensitivity of detection of antibody-antigen interactions using conventional single-particle EM and EMPEM. By combining these two techniques, we leveraged this strategy in different ways to detect the epitopes of low-abundance and low-affinity antibodies that bind to different viral antigens, such as HIV glycoprotein, influenza HAs, and coronavirus spike proteins. First, the functionalization of antigens with an SDAD photo-cross-linker increased the level of detection of a monoclonal antibody in low abundance. Second, a low-affinity monoclonal antibody could be detected only in the presence of a photo-cross-linked HIV trimer. This detection enabled us to structurally characterize the epitope-paratope interactions, which had not been previously possible. Third, photo-cross-linking enabled us to determine the intermediate binding states of two monoclonal influenza antibodies. The photo-cross-linking enabled visualization of conformational intermediates induced upon antibody binding. Finally, this strategy increased detection of pAbs in a series of infection and vaccination studies, illustrating a more in-depth portrait of the immune response landscape over time.

One advantage of this photo-cross-linking strategy is its adaptability. For example, photo-cross-linking can be used in conjunction with an array of viral antigens, including HIV glycoprotein, influenza HA, and HCoV spike—and, in principle, it can be extrapolated to any protein and/or glycoprotein. Furthermore, the superior specificity achieved by the SDAD photo-cross-linker results in efficient and specific protein-protein cross-link interactions. For example, in our hands, discrete bands of cross-linked proteins were visible on a gel after photo-cross-linking compared with chemical cross-linkers, which often results in smears on the gel or even unresolvable aggregates.[14] By using UV irradiation, we improved the specificity of cross-linking and can effectively cross-link low-affinity and low-abundance antibodies to corresponding protein antigens. Looking to the future, we believe that the adaptability of this photo-cross-linking technique may be applied to additional protein-protein interactions, such as increasing enzyme-substrate detectability and helping high-resolution structural characterization of unstable complexes via cryo-EM.

Another important advantage of this photo-cross-linking technique is that pre-functionalization of antigens does not interfere with antibody binding. Photo-cross-linking is therefore biologically compatible with different antibody specificities and binding states, as we demonstrated with both monoclonal antibodies and pAbs. Further, the greater specificity of photo-cross-linkers allowed us to determine the conformational changes that an antibody-antigen complex undergoes upon antibody binding. The

Figure 5. Human serum reactivity to HCoV-SARS-2 spikes and influenza hemagglutinin

(A) Longitudinal dot plots of EMPEM responses observed in three subjects, with epitopes targeted by antibodies assessed at days 28 and 56 against HCoV-SARS-2. "Self-reactive" antibodies are shown in cyan, where overlapping densities potentially suggest a complex epitope composed of antibody and NTD. SARS-2 that was cross-linked via Sulfo-SDAD functionalization is presented in the positive y axis direction and labeled "+CL". SARS-2 that was not cross-linked is shown in the negative y axis direction and labeled "−CL". Labels presented on the y axis correspond to epitopes indicated in the figure legend (B).

(B) Dot plots of EMPEM responses observed in the two subjects from the previous HA study (Figure 4E) against seasonal HCoV strains HKU1 and OC43. Axis labels and "+/−CL" notation is the same as in (A). Included is an exemplary composite model of HCoV antibody epitopes with coloring explained in legend.

(C) Summary of structural composite maps from all five donors with their corresponding glycoprotein antigens broken into three categories: (1) post-vaccination, (2) post-infection, and (3) seasonal antibody response. Darker antibody coloring and corresponding circles indicate responses only observed when the Sulfo-SDAD cross-linker was present. Labels "+" and "−" below composite maps correspond to when the cross-linker was or was not present.

use of photo-cross-linking permitted the detection of three intermediate states once the antibody binds the HA trimer. Thus, photo-cross-linking can be used to resolve antibody binding mechanisms that target other glycoproteins such as HIV trimer or HCoV spike. Additionally, our polyclonal imaging approach using EMPEM in combination with photo-cross-linking provided snapshots of the antibody response at different time points after vaccination and infection as well as the determination of epitopes targeted by pre-existing immune responses. Using this approach, we discerned differences in the kinetics of an antibody response that were correlated with detection of antibody specificities by EMPEM. The addition of photo-cross-linking to this established methodology enabled us to obtain antigen-antibody complexes at early and late time points when the antibody abundance is low. This method could therefore be used for rapid assessment of pre-existing antibody specificities from a patient before vaccination. Altogether, our photo-cross-linking approach can now be deployed for a wide variety of ongoing rational vaccine design efforts.

Limitations of the study

The efficiency of the Sulfo-SDAD photo-cross-linker is limited due to the aggregation of the viral glycoproteins at high molar excess of photo-cross-linker. The efficiency that we achieved using the maximum molar excess of photo-cross-linker to avoid aggregation is ~60%. The goal of our study is to detect low-abundance and low-affinity antibodies in sera and intermediate states of antigen-antibody interactions. Therefore, the efficiency of the cross-linking allowed us to detect these antibodies but might involve the collection of more NS micrographs and particles to increase the number of cross-linked antigen-antibody particles. Additional photo-cross-linkers need to be developed and screened to improve the efficiency of photo-cross-linkers.

The method we developed could be applied to detect antigen-antibody interactions in polyclonal settings using viral glycoproteins from other viruses such as Lassa, Ebola, or respiratory syncytial virus (RSV). Additionally, we have confirmed that this method can detect intermediate states of antigen-antibody interactions upon antibody binding when using monoclonal antibodies. A potential utility of this method could be to determine intermediate states of antigen-antibody in polyclonal sera samples.

STAR★METHODS

Detailed methods are provided in the online version of this paper and include the following:

- KEY RESOURCES TABLE
- RESOURCE AVAILABILITY
  - Lead contact
  - Materials availability
  - Data and code availability
- EXPERIMENTAL MODEL AND STUDY PARTICIPANT DETAILS
  - Rabbit HIV study details
  - Human flu study details
  - CoV study details
  - Cell line details for protein production
- METHOD DETAILS
  - DNA vectors and cloning
  - Protein expression and purification (HIV glycoprotein, H1, H3, OC43, HKU1 and SARS-CoV-2)
  - Human and rabbit samples used in the study
  - Cross-linking glycoproteins and monoclonal Fab-glycoprotein complexes
  - Monoclonal Fab-glycoprotein complexes
  - SDS-PAGE and blue Native-PAGE (BN-PAGE)
  - Amine assay
  - Serum IgG isolation and Fab digestion
  - Purification of antigen-Fab complexes
  - ELISA
  - BLI/OCTET
  - NS-EM data collection
  - Data processing
- QUANTIFICATION AND STATISTICAL ANALYSIS

SUPPLEMENTAL INFORMATION

Supplemental information can be found online at https://doi.org/10.1016/j.crmeth.2023.100509.

ACKNOWLEDGMENTS

We thank B. Anderson, H.L. Turner, and M. Wu for EM data collection support and C. Bowman and J.C. Ducom for computational support. We thank C. Bowman and L. Bond for statistical support. We thank J. Crowe for donating the H5.31 and H5.31 antibodies. We thank D. Burton for the donation of antibodies H10 and A03. This work was supported by NIH NIAID grants AI089992 (A.C.S. and A.B.W.) and AI136621 (A.B.W.) and by the Bill and Melinda Gates Foundation INV-002916 (A.B.W.). We are thankful for the Netherlands Organization for Scientific Research (NWO) Rubicon Grant 45219118 to A.T.d.l.P. and to the NSF Graduate Research Fellowship and Scripps Research Institute Skaggs Graduate School for the David C. Fairchild Endowed Fellowship to L.M.S.

AUTHOR CONTRIBUTIONS

A.T.d.l.P. and L.M.S performed protein purification; ELISA, BLI, and NS-EM experiments; and data processing for the optimization of the photo-cross-linking protocol for the several antigens and monoclonal and polyclonal samples. R.d.P.F.R. performed NS-EM data processing. A.M.J. and S.B. provided samples and performed experiments for SARS-2 antigen. P.P.P. and C.A.C. provided samples and performed NS-EM experiments for HIV EMPEM. S.M. and A.C.S. provided influenza sera samples and HAI titers. A.T.d.l.P., L.M.S., and A.B.W. wrote the paper. All authors contributed to the manuscript text by assisting in writing or providing feedback. A.B.W. supervised the research.

DECLARATION OF INTERESTS

A.B.W. is an inventor on the US patent application no. US 17/536,89 and US 63/154,447, describing the use of EMPEM to map antibody epitopes.

INCLUSION AND DIVERSITY

One or more of the authors of this paper self-identifies as an underrepresented ethnic minority in their field of research or within their geographical location. One or more of the authors of this paper self-identifies as a member of the LGBTQIA+ community. We avoided "helicopter science" practices by including the participating local contributors from the region where we conducted the research as authors on the paper.

CellPress
OPEN ACCESS

Received: October 10, 2022
Revised: April 12, 2023
Accepted: May 30, 2023
Published: June 5, 2023

REFERENCES

1. Ward, A.B., and Wilson, I.A. (2020). Innovations in structure-based antigen design and immune monitoring for next generation vaccines. Curr. Opin. Immunol. *65*, 50–56. https://doi.org/10.1016/j.coi.2020.03.013.

2. Tiller, T., Meffre, E., Yurasov, S., Tsuiji, M., Nussenzweig, M.C., and Wardemann, H. (2008). Efficient generation of monoclonal antibodies from single human B cells by single cell RT-PCR and expression vector cloning. J. Immunol. Methods *329*, 112–124. https://doi.org/10.1016/j.jim.2007.09.017.

3. Walker, L.M., Bowley, D.R., and Burton, D.R. (2009). Efficient recovery of high-affinity antibodies from a single-chain Fab yeast display library. J. Mol. Biol. *389*, 365–375. https://doi.org/10.1016/j.jmb.2009.04.019.

4. Scheid, J.F., Mouquet, H., Feldhahn, N., Seaman, M.S., Velinzon, K., Pietzsch, J., Ott, R.G., Anthony, R.M., Zebroski, H., Hurley, A., et al. (2009). Broad diversity of neutralizing antibodies isolated from memory B cells in HIV-infected individuals. Nature *458*, 636–640. https://doi.org/10.1038/nature07930.

5. Bangaru, S., Antanasijevic, A., Kose, N., Sewall, L.M., Jackson, A.M., Suryadevara, N., Zhan, X., Torres, J.L., Copps, J., de la Peña, A.T., et al. (2021). Structural mapping of antibody landscapes to human betacoronavirus spike proteins. Preprint at bioRxiv. https://doi.org/10.1101/2021.09.30.462459.

6. Antanasijevic, A., Sewall, L.M., Cottrell, C.A., Carnathan, D.G., Jimenez, L.E., Ngo, J.T., Silverman, J.B., Groschel, B., Georgeson, E., Bhiman, J., et al. (2021). Polyclonal antibody responses to HIV Env immunogens resolved using cryoEM. Nat. Commun. *12*, 4817. https://doi.org/10.1038/s41467-021-25087-4.

7. Barnes, C.O., West, A.P., Jr., Huey-Tubman, K.E., Hoffmann, M.A.G., Sharaf, N.G., Hoffman, P.R., Koranda, N., Gristick, H.B., Gaebler, C., Muecksch, F., et al. (2020). Structures of human antibodies bound to SARS-CoV-2 spike reveal common epitopes and recurrent features of antibodies. Cell *182*, 828–842.e16. https://doi.org/10.1016/j.cell.2020.06.025.

8. Bianchi, M., Turner, H.L., Nogal, B., Cottrell, C.A., Oyen, D., Pauthner, M., Bastidas, R., Nedellec, R., McCoy, L.E., Wilson, I.A., et al. (2018). Electron-microscopy-based epitope mapping defines specificities of polyclonal antibodies elicited during HIV-1 BG505 envelope trimer immunization. Immunity *49*, 288–300.e8. https://doi.org/10.1016/j.immuni.2018.07.009.

9. Cottrell, C.A., van Schooten, J., Bowman, C.A., Yuan, M., Oyen, D., Shin, M., Morpurgo, R., van der Woude, P., van Breemen, M., Torres, J.L., et al. (2020). Mapping the immunogenic landscape of near-native HIV-1 envelope trimers in non-human primates. PLoS Pathog. *16*, e1008753. https://doi.org/10.1371/journal.ppat.1008753.

10. van Schooten, J., van Haaren, M.M., Li, H., McCoy, L.E., Havenar-Daughton, C., Cottrell, C.A., Burger, J.A., van der Woude, P., Helgers, L.C., Tomris, I., et al. (2021). Antibody responses induced by SHIV infection are more focused than those induced by soluble native HIV-1 envelope trimers in non-human primates. PLoS Pathog. *17*, e1009736. https://doi.org/10.1371/journal.ppat.1009736.

11. Kumar Bharathkar, S., Parker, B.W., Malyutin, A.G., Haloi, N., Huey-Tubman, K.E., Tajkhorshid, E., and Stadtmueller, B.M. (2020). The structures of secretory and dimeric immunoglobulin A. Elife *9*, e56098. https://doi.org/10.7554/elife.56098.

12. Sharp, T.H., Boyle, A.L., Diebolder, C.A., Kros, A., Koster, A.J., and Gros, P. (2019). Insights into IgM-mediated complement activation based on in situ structures of IgM-C1-C4b. Proc. Nat. Acad. Sci. USA *116*, 11900–11905. https://doi.org/10.1073/pnas.1901841116.

13. Schiffner, T., Pallesen, J., Russell, R.A., Dodd, J., de Val, N., LaBranche, C.C., Montefiori, D., Tomaras, G.D., Shen, X., Harris, S.L., et al. (2018). Structural and immunologic correlates of chemically stabilized HIV-1 envelope glycoproteins. PLoS Pathog. *14*, e1006986. https://doi.org/10.1371/journal.ppat.1006986.

14. Schiffner, T., de Val, N., Russell, R.A., de Taeye, S.W., de la Peña, A.T., Ozorowski, G., Kim, H.J., Nieusma, T., Brod, F., Cupo, A., et al. (2016). Chemical cross-linking stabilizes native-like HIV-1 envelope glycoprotein trimer antigens. J. Virol. *90*, 813–828. https://doi.org/10.1128/jvi.01942-15.

15. Rappuoli, R. (1994). Toxin inactivation and antigen stabilization: two different uses of formaldehyde. Vaccine *12*, 579–581. https://doi.org/10.1016/0264-410x(94)90259-3.

16. Wilton, T., Dunn, G., Eastwood, D., Minor, P.D., and Martin, J. (2014). Effect of formaldehyde inactivation on poliovirus. J. Virol. *88*, 11955–11964. https://doi.org/10.1128/jvi.01809-14.

17. Krammer, F., and Palese, P. (2015). Advances in the development of influenza virus vaccines. Nat. Rev. Drug Discov. *14*, 167–182. https://doi.org/10.1038/nrd4529.

18. Tanaka, Y., and Kohler, J.J. (2008). Photoactivatable crosslinking sugars for capturing glycoprotein interactions. J. Am. Chem. Soc. *130*, 3278–3279. https://doi.org/10.1021/ja7109772.

19. Suchanek, M., Radzikowska, A., and Thiele, C. (2005). Photo-leucine and photo-methionine allow identification of protein-protein interactions in living cells. Nat. Methods *2*, 261–267. https://doi.org/10.1038/nmeth752.

20. Sanders, R.W., Derking, R., Cupo, A., Julien, J.P., Yasmeen, A., de Val, N., Kim, H.J., Blattner, C., de la Peña, A.T., Korzun, J., et al. (2013). A next-generation cleaved, soluble HIV-1 Env trimer, BG505 SOSIP.664 gp140, expresses multiple epitopes for broadly neutralizing but not non-neutralizing antibodies. PLoS Pathog. *9*, e1003618. https://doi.org/10.1371/journal.ppat.1003618.

21. Derking, R., Ozorowski, G., Sliepen, K., Yasmeen, A., Cupo, A., Torres, J.L., Julien, J.P., Lee, J.H., van Montfort, T., de Taeye, S.W., et al. (2015). Comprehensive antigenic map of a cleaved soluble HIV-1 envelope trimer. PLoS Pathog. *11*, e1004767. https://doi.org/10.1371/journal.ppat.1004767.

22. Nogal, B., McCoy, L.E., van Gils, M.J., Cottrell, C.A., Voss, J.E., Andrabi, R., Pauthner, M., Liang, C.-H., Messmer, T., Nedellec, R., et al. (2020). HIV envelope trimer-elicited autologous neutralizing antibodies bind a region overlapping the N332 glycan supersite. Sci. Adv. *6*, eaba0512. https://doi.org/10.1126/sciadv.aba0512.

23. Turner, H.L., Andrabi, R., Cottrell, C.A., Richey, S.T., Song, G., Callaghan, S., Anzanello, F., Moyer, T.J., Abraham, W., Melo, M., et al. (2021). Disassembly of HIV envelope glycoprotein trimer immunogens is driven by antibodies elicited via immunization. Sci. Adv. *7*, eabh2791. https://doi.org/10.1126/sciadv.abh2791.

24. Zost, S.J., Dong, J., Gilchuk, I.M., Gilchuk, P., Thornburg, N.J., Bangaru, S., Kose, N., Finn, J.A., Bombardi, R., Soto, C., et al. (2021). Canonical features of human antibodies recognizing the influenza hemagglutinin trimer interface. J. Clin. Invest. *131*, e146791. https://doi.org/10.1172/jci146791.

25. Bangaru, S., Lang, S., Schotsaert, M., Vanderven, H.A., Zhu, X., Kose, N., Bombardi, R., Finn, J.A., Kent, S.J., Gilchuk, P., et al. (2019). A site of vulnerability on the influenza virus hemagglutinin head domain trimer interface. Cell *177*, 1136–1152.e18. https://doi.org/10.1016/j.cell.2019.04.011.

26. Turner, H.L., Pallesen, J., Lang, S., Bangaru, S., Urata, S., Li, S., Cottrell, C.A., Bowman, C.A., Crowe, J.E., Jr., Wilson, I.A., et al. (2019). Potent anti-influenza H7 human monoclonal antibody induces separation of hemagglutinin receptor-binding head domains. PLoS Biol. *17*, e3000139. https://doi.org/10.1371/journal.pbio.3000139.

27. Bajic, G., Maron, M.J., Adachi, Y., Onodera, T., McCarthy, K.R., McGee, C.E., Sempowski, G.D., Takahashi, Y., Kelsoe, G., Kuraoka, M., et al. (2019). Influenza antigen engineering focuses immune responses to a subdominant but broadly protective viral epitope. Cell Host Microbe *25*, 827–835.e6. https://doi.org/10.1016/j.chom.2019.04.003.

28. McCarthy, K.R., Watanabe, A., Kuraoka, M., Do, K.T., McGee, C.E., Sempowski, G.D., Kepler, T.B., Schmidt, A.G., Kelsoe, G., and Harrison, S.C. (2018). Memory B cells that cross-react with group 1 and group 2 influenza A viruses are abundant in adult human repertoires. Immunity *48*, 174–184.e9. https://doi.org/10.1016/j.immuni.2017.12.009.

29. Tas, J.M.J., Koo, J.H., Lin, Y.C., Xie, Z., Steichen, J.M., Jackson, A.M., Hauser, B.M., Wang, X., Cottrell, C.A., Torres, J.L., et al. (2022). Antibodies from primary humoral responses modulate the recruitment of naive B cells during secondary responses. Immunity *55*, 1856–1871.e6. https://doi.org/10.1016/j.immuni.2022.07.020.

30. Julien, J.P., Cupo, A., Sok, D., Stanfield, R.L., Lyumkis, D., Deller, M.C., Klasse, P.J., Burton, D.R., Sanders, R.W., Moore, J.P., et al. (2013). Crystal structure of a soluble cleaved HIV-1 envelope trimer. Science *342*, 1477–1483. https://doi.org/10.1126/science.1245625.

31. Lyumkis, D., Julien, J.P., de Val, N., Cupo, A., Potter, C.S., Klasse, P.J., Burton, D.R., Sanders, R.W., Moore, J.P., Carragher, B., et al. (2013). Cryo-EM structure of a fully glycosylated soluble cleaved HIV-1 envelope trimer. Science *342*, 1484–1490. https://doi.org/10.1126/science.1245627.

32. Lee, J.H., Leaman, D.P., Kim, A.S., Torrents de la Peña, A., Sliepen, K., Yasmeen, A., Derking, R., Ramos, A., de Taeye, S.W., Ozorowski, G., et al. (2015). Antibodies to a conformational epitope on gp41 neutralize HIV-1 by destabilizing the Env spike. Nat. Commun. *6*, 8167. https://doi.org/10.1038/ncomms9167.

33. Huang, J., Kang, B.H., Pancera, M., Lee, J.H., Tong, T., Feng, Y., Imamichi, H., Georgiev, I.S., Chuang, G.Y., Druz, A., et al. (2014). Broad and potent HIV-1 neutralization by a human antibody that binds the gp41-gp120 interface. Nature *515*, 138–142. https://doi.org/10.1038/nature13601.

34. Lee, J.H., Andrabi, R., Su, C.Y., Yasmeen, A., Julien, J.P., Kong, L., Wu, N.C., McBride, R., Sok, D., Pauthner, M., et al. (2017). A broadly neutralizing antibody targets the dynamic HIV envelope trimer apex via a long, rigidified, and anionic β-hairpin structure. Immunity *46*, 690–702. https://doi.org/10.1016/j.immuni.2017.03.017.

35. Blattner, C., Lee, J.H., Sliepen, K., Derking, R., Falkowska, E., de la Peña, A.T., Cupo, A., Julien, J.P., van Gils, M., Lee, P.S., et al. (2014). Structural delineation of a quaternary, cleavage-dependent epitope at the gp41-gp120 interface on intact HIV-1 Env trimers. Immunity *40*, 669–680. https://doi.org/10.1016/j.immuni.2014.04.008.

36. Feng, M., Bell, D.R., Kang, H., Shao, Q., and Zhou, R. (2019). Exploration of HIV-1 fusion peptide-antibody VRC34.01 binding reveals fundamental neutralization sites. Phys. Chem. Chem. Phys. *21*, 18569–18576. https://doi.org/10.1039/c9cp02909e.

37. Ekiert, D.C., Bhabha, G., Elsliger, M.A., Friesen, R.H., Jongeneelen, M., Throsby, M., Goudsmit, J., and Wilson, I.A. (2009). Antibody recognition of a highly conserved influenza virus epitope. Science *324*, 246–251. https://doi.org/10.1126/science.1171491.

38. Dreyfus, C., Laursen, N.S., Kwaks, T., Zuijdgeest, D., Khayat, R., Ekiert, D.C., Lee, J.H., Metlagel, Z., Bujny, M.V., Jongeneelen, M., et al. (2012). Highly conserved protective epitopes on influenza B viruses. Science *337*, 1343–1348. https://doi.org/10.1126/science.1222908.

39. Rogers, T.F., Zhao, F., Huang, D., Beutler, N., Burns, A., He, W.T., Limbo, O., Smith, C., Song, G., Woehl, J., et al. (2020). Isolation of potent SARS-CoV-2 neutralizing antibodies and protection from disease in a small animal model. Science *369*, 956–963. https://doi.org/10.1126/science.abc7520.

40. Torrents de la Peña, A., Julien, J.P., de Taeye, S.W., Garces, F., Guttman, M., Ozorowski, G., Pritchard, L.K., Behrens, A.J., Go, E.P., Burger, J.A., et al. (2017). Improving the immunogenicity of native-like HIV-1 envelope trimers by hyperstabilization. Cell Rep *20*, 1805–1817. https://doi.org/10.1016/j.celrep.2017.07.077.

41. Bangaru, S., Antanasijevic, A., Kose, N., Sewall, L.M., Jackson, A.M., Suryadevara, N., Zhan, X., Torres, J.L., Copps, J., de la Peña, A.T., et al. (2022). Structural mapping of antibody landscapes to human betacoronavirus spike proteins. Sci. Adv. *8*, eabn2911. https://doi.org/10.1126/sciadv.abn2911.

42. Pettersen, E.F., Goddard, T.D., Huang, C.C., Couch, G.S., Greenblatt, D.M., Meng, E.C., and Ferrin, T.E. (2004). UCSF Chimera—a visualization system for exploratory research and analysis. J. Comput. Chem. *25*, 1605–1612. https://doi.org/10.1002/jcc.20084.

43. Lander, G.C., Stagg, S.M., Voss, N.R., Cheng, A., Fellmann, D., Pulokas, J., Yoshioka, C., Irving, C., Mulder, A., Lau, P.W., et al. (2009). Appion: an integrated, database-driven pipeline to facilitate EM image processing. J. Struct. Biol. *166*, 95–102. https://doi.org/10.1016/j.jsb.2009.01.002.

44. Suloway, C., Pulokas, J., Fellmann, D., Cheng, A., Guerra, F., Quispe, J., Stagg, S., Potter, C.S., and Carragher, B. (2005). Automated molecular microscopy: the new Leginon system. J. Struct. Biol. *151*, 41–60. https://doi.org/10.1016/j.jsb.2005.03.010.

45. Voss, N.R., Yoshioka, C.K., Radermacher, M., Potter, C.S., and Carragher, B. (2009). DoG Picker and TiltPicker: software tools to facilitate particle selection in single particle electron microscopy. J. Struct. Biol. *166*, 205–213. https://doi.org/10.1016/j.jsb.2009.01.004.

46. Scheres, S.H. (2012). RELION: Implementation of a Bayesian approach to cryo-EM structure determination. J. Struct. Biol. *180*, 519–530. https://doi.org/10.1016/j.jsb.2012.09.006.

47. Punjani, A., Rubinstein, J.L., Fleet, D.J., and Brubaker, M.A. (2017). cryoSPARC: algorithms for rapid unsupervised cryo-EM structure determination. Nat. Methods *14*, 290–296. https://doi.org/10.1038/nmeth.4169.

48. Schneider, C.A., Rasband, W.S., and Eliceiri, K.W. (2012). NIH Image to ImageJ: 25 years of image analysis. Nat. Methods *9*, 671–675. https://doi.org/10.1038/nmeth.2089.

49. Binley, J.M., Sanders, R.W., Clas, B., Schuelke, N., Master, A., Guo, Y., Kajumo, F., Anselma, D.J., Maddon, P.J., Olson, W.C., et al. (2000). A recombinant human immunodeficiency virus type 1 envelope glycoprotein complex stabilized by an intermolecular disulfide bond between the gp120 and gp41 subunits is an antigenic mimic of the trimeric virion-associated structure. J. Virol. *74*, 627–643. https://doi.org/10.1128/jvi.74.2.627-643.2000.

50. Pugach, P., Ozorowski, G., Cupo, A., Ringe, R., Yasmeen, A., de Val, N., Derking, R., Kim, H.J., Korzun, J., Golabek, M., et al. (2015). A native-like SOSIP.664 trimer based on an HIV-1 subtype B env gene. J. Virol. *89*, 3380–3395. https://doi.org/10.1128/jvi.03473-14.

51. Pettersen, E.F., Goddard, T.D., Huang, C.C., Meng, E.C., Couch, G.S., Croll, T.I., Morris, J.H., and Ferrin, T.E. (2021). UCSF ChimeraX: structure visualization for researchers, educators, and developers. Protein Sci. *30*, 70–82. https://doi.org/10.1002/pro.3943.

STAR★METHODS

KEY RESOURCES TABLE

REAGENT or RESOURCE	SOURCE	IDENTIFIER
**Antibodies**		
2G12	Produced in house (Sanders et al.[20])	RRID:AB_2819235
PGT122	Produced in house (Julien et al., 2013[30])	RRID:AB_2491042
PGT128	Produced in house (Julien et al., 2013[30])	RRID:AB_2491047
3bnc117	Produced in house (Lyumkis et al., 2013[31])	RRID:AB_2491033
3BC315	Produced in house (Lee et al., 2015[32])	N/A
35O22	Produced in house (Huang et al., 2014[33])	N/A
PG9	Produced in house (Julien et al., 2013[30])	RRID:AB_2491030
PGT145	Produced in house (Lee et al., 2017[34])	RRID:AB_2491054
PGT151	Produced in house (Blattner et al., 2014[35])	N/A
VRC034	Produced in house (Feng et al., 2019[36])	N/A
14e	Produced in house (Sanders et al.[20])	N/A
19b	Produced in house (Sanders et al.[20])	N/A
CR6261	Produced in house (Ekiert et al., 2009[37])	N/A
CR9114	Produced in house (Dreyfus et al., 2012[38])	N/A
H10	Collaboration and donation of antibodies (Rogers et al., 2020[39])	N/A
A03	Collaboration and donation of antibodies (Rogers et al., 2020[39])	N/A
H5.28	Produced in house (Turner et al.[26])	N/A
H5.31	Produced in house (Turner et al.[26])	N/A
RM19D	Produced in house (Cottrell et al.[9])	N/A
**Biological samples**		
Plasma or serum from human, immunized with influenza vaccine	Yale School of Medicine (Dr. Albert C. Shaw)	0409027018
Plasma or serum from rabbits, immunized with HIV BG505 glycoprotein	Cottrell et al.[9]	IACUC 14-0002
Plasma or serum from human, infected with CoV-SARS-2	Vanderbilt University School of Medicine (Dr. James E. Crowe)	N/A
**Chemicals, peptides, and recombinant proteins**		
BG505 SOSIP.664	Produced in house (Sanders et al.[20])	N/A
BG505 SOSIP.v5.2	Produced in house (Torrents de la Peña et al., 2017[40])	N/A
H1 A/Mich/045/15	Produced in house (fludb.org)	N/A
H3 A/Sing/INFIMH/16	Produced in house (fludb.org)	N/A
OC43	Produced in house (fludb.org)	N/A
HKU1	Produced in house (fludb.org)	N/A
SARS-2	Produced in house (fludb.org)	N/A
H1 A/Ca/09	Produced in house (fludb.org)	N/A
Sulfo-SDA	Sigma Aldrich	Cat #803340
Sulfo-LC-SDA	Sigma Aldrich	Cat #803359
Sulfo-SDAD	Sigma Aldrich	Cat #803367
Superose 200 Increase Size Exclusion Column	Sigma Aldrich	Cat #GE28-9909-44
papain	Sigma Aldrich	Cat # 9001-73-4

(Continued on next page)

Continued

REAGENT or RESOURCE	SOURCE	IDENTIFIER
Native-PAGE gels and buffer	Thermo Fisher	Cat # NP032A
SDS-PAGE gels and buffer	ThermoFisher	Cat #XP04125BOX
Octet FAB2G sensors	Sartorius	Cat # 18-5125
Uranyl formate	Electron Microscopy Sciences	Cat# D310 25 GM
CaptureSelect™ IgG-Fc (Multispecies) Affinity Matrix	Thermo Fisher	Cat #19431801L
**Deposited data**		
nsEM maps of subject 182419 over time complexed with H1	This paper	EMD-27346 to EMD-27354
nsEM maps of subject 182419 over time complexed with H3	This paper	EMD-27355 to EMD-27365
nsEM maps of subject 182419 over time complexed with HCoV spikes	This paper	EMD-27366 to EMD-27369
nsEM maps of subject 182322 over time complexed with H1	This paper	EMD-27370 and EMD-27371
nsEM maps of subject 182322 over time complexed with H3	This paper	EMD-27372 and EMD-27373
nsEM maps of subject 182322 over time complexed with HCoV spikes	This paper	EMD-27374 to EMD-27377
nsEM maps of influenza H1 complexed with monoclonal antibody H5.28 and H5.31 Fab	This paper	EMD-27378 and EMD-27379
nsEM maps of BG505 SOSIP complexed with monoclonal antibody 3E1	This paper	EMD-27380
nsEM maps of rabbit T8640 complexed with polyclonal Fab at week 18	This paper	EMD-27381
**Experimental models: Cell lines**		
Human: FreeStyle HEK293F	Thermo Fisher Scientific	Cat# R79007
**Oligonucleotides**		
ctccggcggatctagcgccTGGTCCCACCCCCAGTTC	IDT	https://idtdna.com/
cctccgccgctgccgccgccCTTCTCAAATTGAGGGT GAGACCAG	IDT	https://idtdna.com/
**Recombinant DNA**		
BG505 SOSIP.664	(Sanders et al.[20])	N/A
BG505 SOSIP.v5.2	(Torrents de la Peña et al., 2017[40])	N/A
H1 A/Mich/045/15	Produced in house (fludb.org)	https://genscript.com/
H3 A/Sing/INFIMH/16	Produced in house (fludb.org)	https://genscript.com/
OC43	(Bangaru et al., 2022[41])	N/A
HKU1	(Bangaru et al., 2022[41])	N/A
SARS-2	(Bangaru et al., 2022[41])	N/A
H1 A/Ca/09	(Turner et al.[26])	N/A
**Software and algorithms**		
Unicorn 7.0	GE Healthcare	https://www.gelifesciences.com/
UCSF Chimera	(Pettersen et al.[42])	N/A
Appion database	(Lander et al.[43])	N/A
Leginon	(Suloway et al.[44])	N/A
DoG Picker	(Voss et al., 2009[45])	N/A
Relion	(Scheres, 2012[46])	N/A
CryoSparc	(Punjani et al.[47])	N/A
Prism 9 - Graphpad	Dotmatics	https://www.graphpad.com/features
ImageJ	(Schneider et al., 2012[48])	https://imagej.nih.gov/ij/

RESOURCE AVAILABILITY

Lead contact

Further information and requests for resources and reagents should be directed to and will be fulfilled by the lead contact, Andrew Ward (andrew@scripps.edu).

Materials availability

This study did not generate new unique reagents.

Data and code availability

- The EMDBs have been deposited into the EMDB database (https://www.ebi.ac.uk/emdb) and are publicly available as of the date of publication. The accession numbers are listed in the key resources table.
- This paper does not report original code.
- Any additional information required to reanalyze the data reported in this paper is available from the lead contact upon request.

EXPERIMENTAL MODEL AND STUDY PARTICIPANT DETAILS

Rabbit HIV study details

One serum sample from the study IACUC 14-0002 from Scripps Research was used for EMPEM. New Zealand White Female rabbits were vaccinated at weeks 0, 4 and 20 and sera from week 2 was used for EMPEM studies. Female rabbits were used for immunization because they elicit a stronger immune response.

Human flu study details

Serum samples from two subjects (182322 and 182419) were used for EMPEM. Subjects were part of the study 0409027018 from Yale University. We do not have data on recruitment and therefore cannot state how sex, gender, or ethnicities were accounted for. Briefly, healthy participants were immunized with high-dose trivalent Fluzone provided by Sanofi Pasteur, Inc. All participants provided informed consent. Sera was collected at days 0, 2, 7, 28 and 70 after immunization at day 0.

CoV study details

Serum samples from three donors (1988, 1989, 1992) were used for EMPEM studies. Patient samples have been described previously.[5] The studies were approved by the Institutional Review Board of Vanderbilt University Medical Center. Samples were obtained after written informed consent. We do not have data on recruitment and therefore cannot state how sex, gender, or ethnicities were accounted for.

Cell line details for protein production

HEK293F and FreeStyle 293-F media were purchased from Thermo Fisher Scientific and used following manufacturer suggestions. For further details see below in methods.

METHOD DETAILS

DNA vectors and cloning

BG505 *env* gene is derived from a subtype A virus. The soluble HIV Env construct BG505 SOSIP.v3 has been described elsewhere.[20] It contains a gp120-gp41$_{ECTO}$ disulfide bond at position A501C-T605C[49]; an I559P in gp41$_{ECTO}$; a furin cleavage enhancement in gp120 (REKR to RRRRRR) and a stop codon at gp41$_{ECTO}$ residue 664.[49]

H1 and H3 are derived from A/Michigan/045/15 H1 and A/Singapore/INFIMH/16 H3 *hemagglutinin* genes, respectively. The genes were cloned into pcDNA3.4 vectors using NotI and AgeI restriction enzymes. H1 and H3 contain a foldon trimerization domain (foldon: MKQIEDKIEEILSKIYHIENEIARIKKLIGE) followed by an 8x His-tag (HHHHHHHHH) and a Twin-Strep-tag (WSHPQFEKGGGS GGGSGGSAWSHPQFEK) at the C-terminus of the foldon.

HCoV-OC43, HCoV-HKU1 and HCoV-SARS-2 contain a C-terminal T4 fibritin trimerization domain followed by a HRV3C cleavage site, an 8x His-tag and a Twin-strep-tag. The OC43, HKU1 and SARS spike constructs have been described elsewhere.[5] The OC43 and HKU1 contain stabilizing prolines at residues 1,079 and 1,080, and 1,067 and 1,068, respectively. We generated three mutants of SARS-2 and mixed equal ratios of the mutants in each of the assays. The SARS-2 mutants contain the HexaPro substitution, an S1/S2 furin cleavage and a disulfide mutation at: HP-GSAS Mut2 (S383C and D985C), HP-GSAS Mut4 (A570C and L966C) or HP-GSAS Mut7 (V705C and T883C).[5]

Protein expression and purification (HIV glycoprotein, H1, H3, OC43, HKU1 and SARS-CoV-2)

For protein expression, FreeStyle 293F cells were transfected at a density of 1×10^6 cells/mL as previously described.[20] Briefly, PEI-MAX (1 mg/mL) was mixed with HIV glycoprotein and Furin plasmids at a 4:1 ratio (Env:Furin) in OPTI-MEM. The mixture was added to the cells and cultures were harvested at 6-day post-transfection.

For HIV, Env glycoproteins were purified by affinity chromatography using a PGT145 column as previously described.[50] Supernatants were filtered through 0.45μm filters and passed through the PGT145 column at a flow rate of 1 mL/min. Bound HIV Env glycoproteins were eluted using 1x CV of 3 M $MgCl_2$ prior to buffer exchange to TN75 (75mM NaCl, 10mM Tris, pH 8.0). Trimers were further purified using a Superdex 200 increase 10/300 column (GE Healthcare Biosciences) in TBS buffer.

For HA proteins, the proteins were purified from the supernatants using cOmplete™ His-Tag purification resin (Millipore Sigma). The supernatant was passed through the columns at a 1 mL/min flow rate and the protein was eluted using 250 mM imidazole elution buffer. The purified protein was buffer exchanged to TBS buffer (25mM Tris, 500mM NaCl, pH 7.4) prior to further purification with Superdex 200 increase 10/300 column (GE Healthcare Biosciences) in TBS buffer.

For OC43, HKU1 and SARS, the spike proteins were purified from supernatants using StrepTactin-XT 4FLOW gravity flow columns (IBA Lifesciences). The supernatant was passed through the columns at a flow rate of 1 mL/min. The protein was incubated with BXT elution buffer for 4 h, eluted and further purified using a Superose 6 increase 10/300 column (GE Healthcare Biosciences) in TBS buffer.

All the protein fractions corresponding to the trimeric proteins were collected and concentrated using a 30 kDa cutoff Amicon ultrafiltration units. The quality of the proteins was assessed by Negative Stain EM for further use.

Human and rabbit samples used in the study

For all the assays described in the paper, serum samples for donors 182322, 182419 were used to test HA, OC43 and HKU1, plasma samples for donors 1988, 1989 and 1992 were used for SARS-2 spike proteins and a serum sample for rabbit rt8640 was used for HIV-1 protein.

Cross-linking glycoproteins and monoclonal Fab-glycoprotein complexes

For HIV trimers, first, glutaraldehyde was used to cross-link apo-trimers as previously described (Schiffner et al., 2014).[14] Briefly, HIV trimers at a concentration of 1 mg/mL were mixed with an equal volume of glutaraldehyde. The mixture was incubated for 5min and Tris buffer at pH 8.0 was added to a final concentration of 75mM. After 10 min, the protein was buffer-exchanged to TBS for further analysis. Second, three Succinimidyl-diazirine (SDA) cross-linkers were tested: Sulfo-SDA (sulfosuccinimidyl 4,4′-azipentanoate), Sulfo-LC-SDA (sulfosuccinimidyl 6-(4,4′-azipentanamido)hexanoate) and Sulfo-SDAD (sulfosuccinimidyl 2-[(4,4′-azipentanamido) ethyl]-1,3′-dithiopropionate]) (Sigma Aldrich). Each cross-linker was diluted in PBS to a final concentration of 1mM. BG505 SOSIP.v3 at 10 μM was incubated for 30 min at RT with 50x molar excess of each cross-linker. The reaction was quenched with 75mM Tris:HCl at pH 8.0. for 5 min. The excess of cross-linker was eliminated using a 30kDa cutoff Amicon ultrafiltration units. The trimers were cross-linked using the UVP Crosslinker CL-3000 (AnalitikJena) at 1200 kJ/s for 5 min. Cross-linking efficiency was assessed using SDS-PAGE (described below).

The method was further optimized using Sulfo-SDAD for several glycoproteins (HA, HIV, CoV) based on the protocol described above and using different molar excess amounts of cross-linker (range 0mM–100mM), time of cross-linking (0–15 min) and glycoproteins.

The protocol was optimized as follows: BG505 SOSIP.v3, HA, OC43, HKU1 and SARS-2 were diluted in PBS to a final concentration of 5 μM and mixed with 20x molar excess of Sulfo-SDAD. The reaction was incubated at room temperature for 30 min and stopped with the addition of Tris:HCl pH 8.0 at a final concentration of 75 mM for 5 min. HIV and HCoV trimers were cross-linked using the UVP Crosslinker CL-3000 (analitikJena) at 1200 kJ/s for 5 min while HA trimers were cross-linked at 600 kJ/s for 5 min.

Monoclonal Fab-glycoprotein complexes

For the titration of HIV BG505 SOSIP.v3 trimers in complex with 3bnc117 Fab, trimer-Fab complexes were formed using a range of excess molar ratios of Fab (0.008x – 3x). The complexes were incubated at RT for 15 min prior to being applied to carbon-coated copper grids. The occupancy was assessed by NS-EM.

Additionally, HIV BG505 SOSIP.v3 trimer was cross-linked with multiple monoclonal antibodies. The titration was performed by cross-linking the BG505 SOSIP.v3 antigen with different molar ratios of Fab as follows: BG505 SOSIP.v3 at 5 μM was mixed with 20x molar excess of Sulfo-SDAD. The reaction was incubated at room temperature for 30 min and stopped with the addition of Tris:HCl pH 8.0 at a final concentration of 75 mM for 5 min. The trimer functionalized with Sulfo-SDAD cross-linker was incubated with different amounts of Fab for either 15 min at RT and then cross-linked or immediately cross-linked with UV at 1200 kJ/s for 5 min.

For HA trimers complexed with H5.28 Fab and H5.31 Fab, Ca09 containing the stabilizing mutation E47K at 5 μM was mixed with 20x molar excess of Sulfo-SDAD. The reaction was incubated at room temperature for 30 min and stopped with the addition of Tris:HCl pH 8.0 at a final concentration of 75 mM for 5 min. The HA trimer functionalized with Sulfo-SDAD was incubated with 3x molar excess of Fab and immediately cross-linked at 600 kJ/s for 5 min.

SDS-PAGE and blue Native-PAGE (BN-PAGE)

Proteins were analyzed using SDS-PAGE followed by Coomassie blue dye staining. 2 μg of protein were mixed with loading dye (Genscript) and incubated for 10 min at 95°C prior to loading on a 4–12% Tris-Glycine gel (Invitrogen). For reducing SDS-PAGE, 100mM of dithiothreitol (DTT) was included in the loading mixture. The gels were run at 200V for 35 min using Novex Tris-Glycine SDS Running Buffer (Invitrogen) and stained with InstantBlue™ coomassie stain (AbCam) for 1 h.

For BN-PAGE, 2 μg of protein was mixed with loading dye (Genscript) and directly loaded onto a 4–12% Bis-Tris NuPAGE gel (Invitrogen). The gels were run using Anode-Buffer (Invitrogen) and Cathode-Buffer (Invitrogen) for 1 h at 200V. Proteins from BN-PAGE gels were fixed using fixation solution (40% methanol 10% acetic acid) and the gel was distained with water overnight.

Amine assay

SOSIP or GLA-SOSIP trimer (5 μg) in 20 μL PBS was added to 30 μL of 0.1 M NaHCO3, pH 8.5. 25 μL of 5% 2,4,6-Trinitrobenzene Sulfonic Acid (TNBSA) diluted 1/500 in 0.1 M NaHCO3 pH 8.5 was added to the samples for 2 h at 37°C, followed by 25 μL of 10% SDS and 12.5 μL of 1M HCl. Samples were vortexed and the optical density read at 335 nm. The relative quantity of free amines was calculated as (OD_{335} (GLA-SOSIP trimer)–OD_{335} (blank))/(OD_{335} (SOSIP trimer)–OD_{335} (blank)).

Serum IgG isolation and Fab digestion

CaptureSelect IgG-Fc (Multispecies) Affinity Matrix (Thermo Scientific™) was washed three times with PBS at a 1:25 ratio (ml resin: ml PBS). 1 mL of human serum was mixed with 1 mL of washed CaptureSelect resin and 3.5 mL of PBS. The mixture was incubated for 48–72 h at 4°C. Next, the resin was spun down at 3,500g for 5 min and washed three times with 10 mL of PBS. Polyclonal IgG was eluted by incubating the resin with 10 mL of 0.1 M glycine buffer at pH 2.5 for 30 min. The eluted IgG was immediately neutralized with 4 mL of Tris-HCl, pH 8.0 and buffer exchanged to PBS using 30 kDa cutoff Amicon ultrafiltration units. For Fab digestion, papain was activated for 15 min at 37°C in digestion buffer (100mM Tris, 2mM EDTA, 10mM L-Cysteine, 1 mg/mL papain). Next, 5 mg of polyclonal IgG were incubated in digestion buffer with papain (20 mM sodium phosphate, 10 mM EDTA, 20 mM cysteine, 0.1 mg/mL papain, pH 7.4). The reaction was incubated for 4–5 h at 37°C. Iodacetamide was added to the sample at a final concentration of 0.03 M to quench the reaction. The digested IgG was concentrated and buffer exchanged to TBS pH 7.4 using 10 kDa cutoff Amicon ultrafiltration units. The undigested IgGs were removed by size exclusion chromatography using a Superose 200 increase column (GE Healthcare Biosciences) in TBS buffer. The fractions containing purified Fabs were concentrated using 10 kDa Amicon ultrafiltration units.

Purification of antigen-Fab complexes

Complexes were generated by incubating 15 μg of antigen with 500 μg of Fab for HA, OC43 and HKU1 or 15 μg of antigen with 3–5 mg of Fab for SARS-2 overnight at RT.

For cross-linked complexes, the cross-linker was functionalized to the antigen (HA, OC43, HKU1 or SARS) as previously described. The antigen was incubated with purified polyclonal Fab overnight at RT. For HA, OC43 and HKU1, 15 μg of antigen was then incubated with 500 μg of purified polyclonal Fab, while for SARS-2, 15 μg of antigen were incubated with 3–5 mg of purified Fab. After incubation, antigen-Fab complexes were cross-linked by exposing them to UV for 5 min at 1,200 kJ/s or at 600 kJ/s for OC43, HKU1, SARS and HA, respectively.

The complexes were purified on a Superose 6 increase 10/300 column using UV absorbance at 215 nm on Akta Pure system (GE Healthcare) running in TBS buffer. The fractions containing antigen-Fab complexes were concentrated using 10 kDa cutoff Amicon ultrafiltration units and immediately used for making EM grids.

ELISA

For HA, Microlon-600 96-well, half-area plates (Greiner Bio-One) were coated for an hour with purified HA at 25 μg/mL in 0.1 M $NaHCO_3$, pH 8.6 (50 μL/well). Unbound trimers were removed by 3 wash steps with TBS 0.1% tween 20 before prior to a blocking with PBS 5% BSA overnight. After washing and blocking steps, sera from different timepoints were serially diluted in PBS in 3-fold steps starting at 1:30 dilution. After three washes with TBS 0.1% tween 20, AP-conjugated AffiniPure goat anti-human IgG (Jackson Immunoresearch, Cat # 109-055-097) was added at a 1:5000 dilution in TBS 1% BSA. Colorimetric detection was performed using alkaline phosphatase yellow (pNPP) liquid substrate (Thermo-Fisher Scientific). Color development (absorption at 450 nm) was stopped using 2 M NaOH (25 μL) when a plateau value was reached in the first two wells containing the highest sera concentration. Data was recorded on a Synergy H1 plate reader (BioTek) and curves and midpoint titers were plotted and calculated using Prism version 8.3.0. Experiments were performed in duplicate. Data are represented as mean ± SEM.

BLI/OCTET

BLI assays were performed using the Octet Red96 instrument (Pall FortéBio). IgG was immobilized onto Dip and Read anti-human IgG Quantitation (AHQ) Biosensors for 120 s followed by a 60 s baseline measurement in Kinetics buffer (PBS pH 7.2 with 0.01% w/v bovine serum albumin and 0.002% v/v tween 20). The biosensors were then dipped for 300 s into wells containing BG505 SOSIP.v3 diluted in kinetics buffer at a final concentration of 200nM. The sensors were dipped into wells containing kinetics buffer for 600 s to assess dissociation. Curves were aligned to determine on- and off-rates.

NS-EM data collection

Purified BG505 SOSIP.v3, HA and HCoV trimers, either alone, cross-linked or as antigen-Fab complexes were diluted to 20–30 μg/mL and applied for 10 s to carbon-coated 400 mesh Cu grids, that had been glow discharged at 15 mA for 20 s. For HIV and HA trimers or trimer-Fab complexes the sample was negatively stained with 2% (w/v) uranyl-formate for 40 and 50 s, respectively. For CoV trimers or trimer-Fab complexes the sample was stained for 100 s. Data was collected on a Tecnai Spirit electron microscope operating at 120keV or a Tecnai TF20 electron microscope operating at 200 keV. Nominal magnification was 52,000× and 62,000 X with a pixel size at 2.06 Å and 1.77 Å (at the specimen plane) for the Spirit and TF20, respectively. The nominal defocus range was set between −1.5 and −2 μm and the electron dose was calibrated to 25 $e^-/Å^2$. Micrographs were recorded using a Tietz (4k) TemCam-F416 CMOS or FEI Eagle CCD (4k). Data was acquired using the Leginon automated imaging interface.[44]

Data processing

To assess the quality of cross-linked and non-cross-linked antigens, initial processing was conducted using the Appion data processing package,[43] where approximately 30,000 particles were picked using the automated picking. Particle coordinates were then transferred to Relion/3.0, where particles were extracted and 2D-classified into 50 classes (25 iterations). Particles corresponding to trimers were selected and another round of 2D classification was carried out.

For the titration of BG505 SOSIP.v3 complexed with different molar excess of 3bnc117 Fab, data processing was carried out as described above with minor changes. In this case, approximately 50,000 particles were picked. Another round of 2D classification was performed using particles corresponding to trimers or trimer-Fab complexes. To analyze the interactions and occupancy of the 3bnc117 Fab with BG505 SOSIP.v3, the 2D class averages were examined. One, two or three Fabs were clearly visualized if they were bound to the trimer, allowing the percentage of occupancy relative to unbound trimers to be calculated.

The different bound states of H5.28 Fab with HA were processed as follows. 50,000 particles were automatically picked, extracted and 2D classified as mentioned previously. Particle containing antigen-Fab complexes were selected for 3D analysis. Initial 3D refinement was performed with a low-resolution model of a non-liganded HA trimer to align the particles. 3D classification into 20–40 classes was performed using a low-resolution model of a non-liganded HA monomer. Classes with similar features were combined and reclassified and classes with unique Fab features, representing different bound states, were further processed using 3D refinement. Maps were visualized and segmented using UCSF Chimera 1.13.[51]

The different bound states of H5.31 Fab with HA were processed using the Relion 3.0 and CryoSPARC software packages.[47] Image preprocessing was performed using the Appion image processing package. Particles were selected from the micrographs using the cryoSPARC image processing suite (blob picker). A total of ~191,000 particles were extracted from the micrographs and 2D classified. Approximately 110,000 particles were selected for further processing in 3D. Three rounds of heterogeneous refinement (n = 2, n = 3 and n = 3, respectively) using a low-resolution HA monomer and trimer produced two 3D classes containing particles that represented two different states of HA bound to H5.31 Fab: an HA dimer in complex with two Fabs and an HA monomer in complex with one Fab. The two 3D classes were further processed using non-uniform refinement. Maps were visualized and segmented using UCSF Chimera 1.13.[51]

For antigen complexes with purified polyclonal antibodies, 100,000–200,000 particles were automatically picked using the Appion image processing package. Particles were transferred to Relion/3.0, extracted and 2D-classified into 200 classes. Particles containing trimer only or trimer-Fab complexes were selected for 3D analysis. Initial 3D classification was performed using a minimum of 50,000 particles for HA-Fab complexes and 100,000 particles for CoV-Fab complexes. The 3D reference for all 3D classification and refinements was a low-resolution model of a non-liganded HA or CoV trimer. For HA and CoV complexes, initial 3D Refinement was performed to align all the particles prior to 3D classification, where particles were classified into 20–40 classes. For CoV complexes, particles were classified to 60–100 classes. Subsequently, classes with similar features were combined and reclassified and classes with unique Fab specificities were further processed using 3D refinement. Maps were visualized and segmented using UCSF Chimera 1.13.[42]

QUANTIFICATION AND STATISTICAL ANALYSIS

Statistical analyses were performed using the PRISM software. Median and Mean were calculated and are described in the results section as well as the Figure legends.

Cell Reports Methods, Volume *3*

Supplemental information

Increasing sensitivity of antibody-antigen interactions using photo-cross-linking

Alba Torrents de la Peña, Leigh M. Sewall, Rebeca de Paiva Froes Rocha, Abigail M. Jackson, Payal P. Pratap, Sandhya Bangaru, Christopher A. Cottrell, Subhasis Mohanty, Albert C. Shaw, and Andrew B. Ward

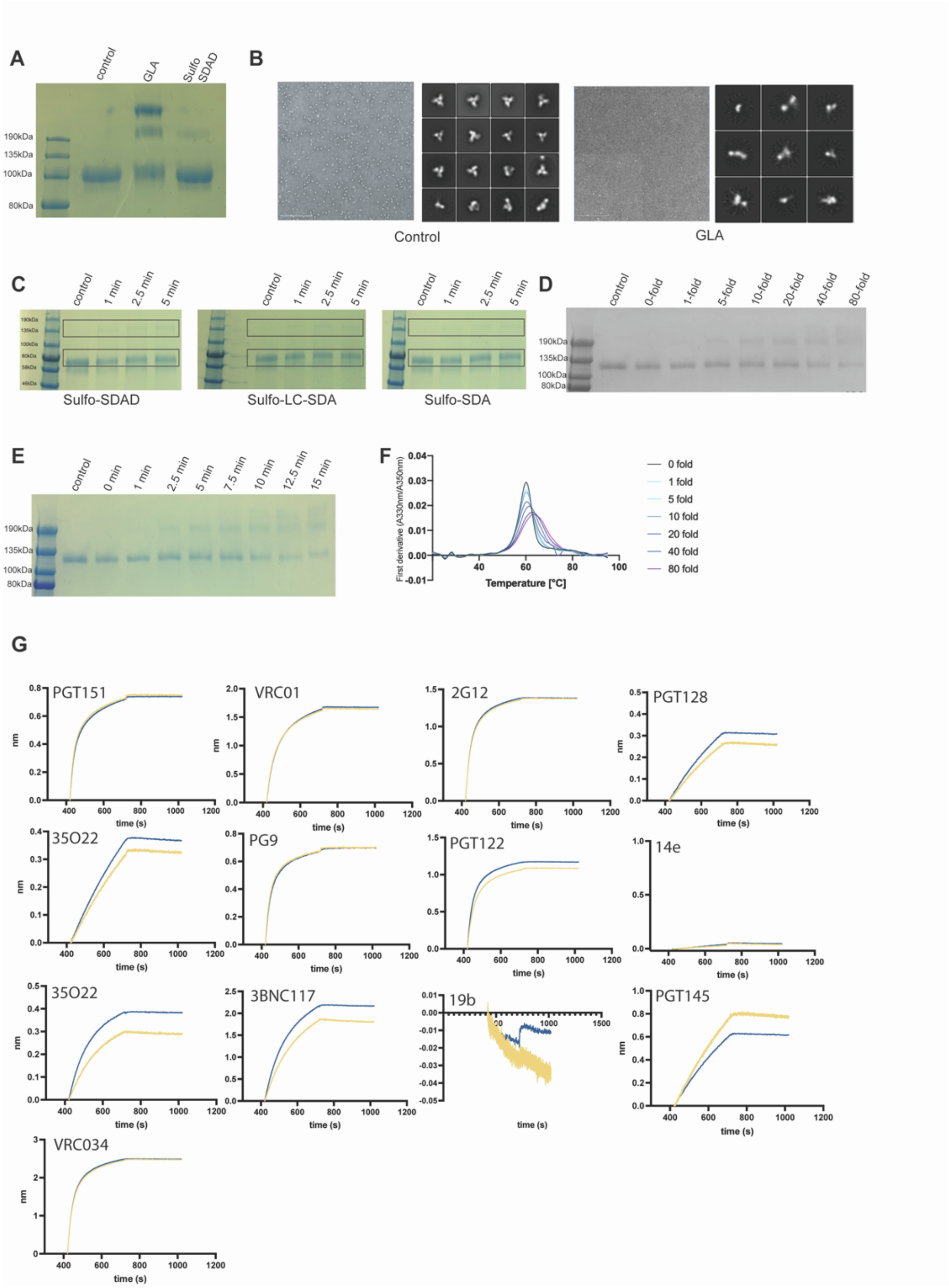

Figure S1. Screening and optimizing photo-cross-linkers using the BG505 HIV trimer. Related to Figure 1. (**A**) Non-reduced SDS-PAGE of HIV trimer using GLA and 20x fold molar excess of photo-cross-linker Sulfo-SDAD. (**B**) NS-EM micrograph and 2D classes of non-cross-linked and GLA cross-linked BG505 SOSIP.v3. (**C**) Non-reduced SDS-PAGE of HIV trimer using 20x fold molar excess of three photo-cross-linkers (Sulfo-SDAD, Sulfo-LC-SDA and Sulfo-SDA) and three UV exposure lengths: 1 min, 2.5 min and 5 min. (**D-E**) Sulfo-SDAD was used to further proceed for cross-linking. A molar range of cross-linker (panel B) and a time course UV irradiation using Sulfo-SDAD (panel C) were used to optimized cross-linking. (**F**) Nano-DSF

analysis of several cross-linker molar excess conditions was visualized by plotting the first derivative. The T_m values are listed in Fig 1. (**G**) Antigenicity was assessed by using BLI and by testing binding of a set of antibodies to the cross-linked HIV trimer or the HIV trimer alone. The antibody panel includes broadly neutralizing antibodies and non-neutralizing antibodies targeting epitopes located at different regions of the HIV trimer.

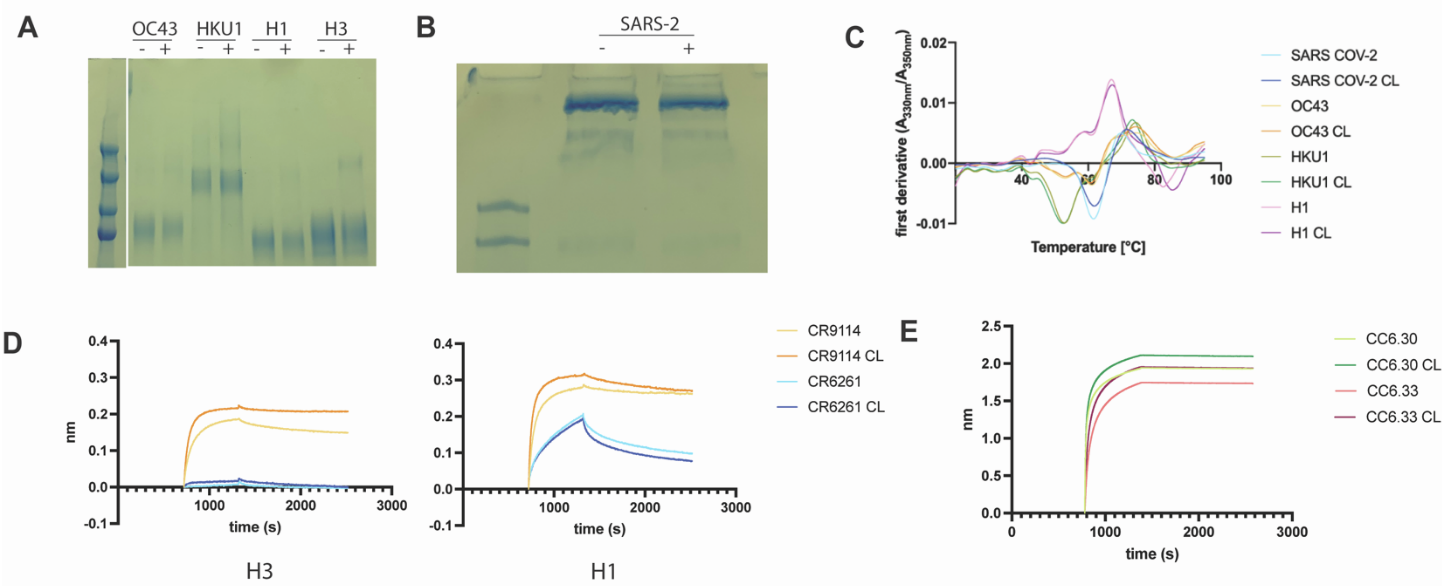

Figure S2. Biochemical and biophysical properties of cross-linked hemagglutinin and HCoV spikes.

Related to Figure 2. (**A**) Non-reduced SDS-PAGE of seasonal HCoV variants OC43 and HKU1, influenza hemagglutinin H1 and H3 and (**B**) HCoV SARS-2. (**C**) Nano-DSF analysis of HCoV and influenza HA variants is plotted. The T_m values are listed in Fig 2. (**D and E**) Antigenicity of influenza HA (D) and SARS-2 (E) was assessed by BLI using two broadly neutralizing antibodies against the HA stem (CR9114 and CR6261) and the SARS-2 RBD (CC6.30 and CC6.33), respectively.

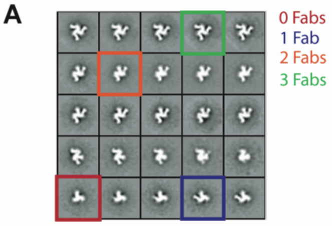

B

	Fab molar excess	number of particles	Frequency of Fab occupancy 0 Fab	1 Fab	2 Fab	3 Fab
CL	**0.004**	34,856	1	0	0	0
	**0.008**	40,675	1	0	0	0
	**0.016**	34,456	0.99	0.01	0	0
	**0.031**	65,195	0.96	0.04	0	0
	**0.065**	44,081	0.91	0.07	0.02	0
	**0.125**	21,411	0.83	0.15	0.01	0
	**0.25**	55,555	0.65	0.19	0.12	0.04
	**0.5**	45,221	0.43	0.29	0.22	0.06
	**1**	43,076	0.31	0.18	0.27	0.26
	**3**	50,046	0	0.01	0.3	0.69
non-CL	**0.004**	45,442	1	0	0	0
	**0.008**	39,166	1	0	0	0
	**0.016**	38,019	1	0	0	0
	**0.031**	53,407	0.98	0.02	0	0
	**0.065**	28,573	0.96	0.02	0	0
	**0.125**	25,765	0.88	0.11	0.01	0
	**0.25**	29,345	0.76	0.17	0.05	0.01
	**0.5**	52,120	0.44	0.24	0.23	0.09
	**1**	68,237	0.23	0.25	0.3	0.22
	**3**	61,186	0.03	0	0.01	0.96

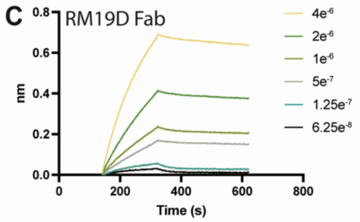

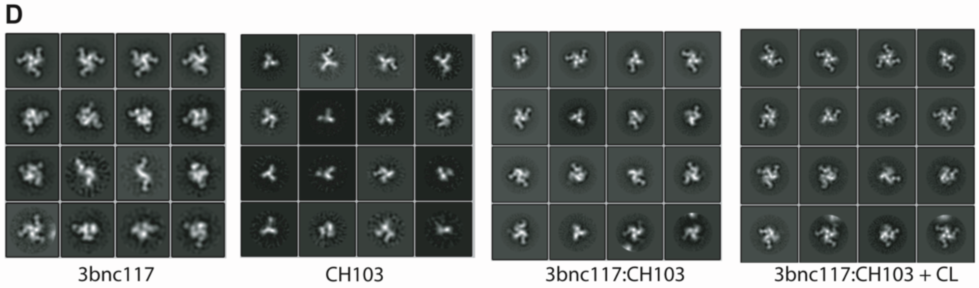

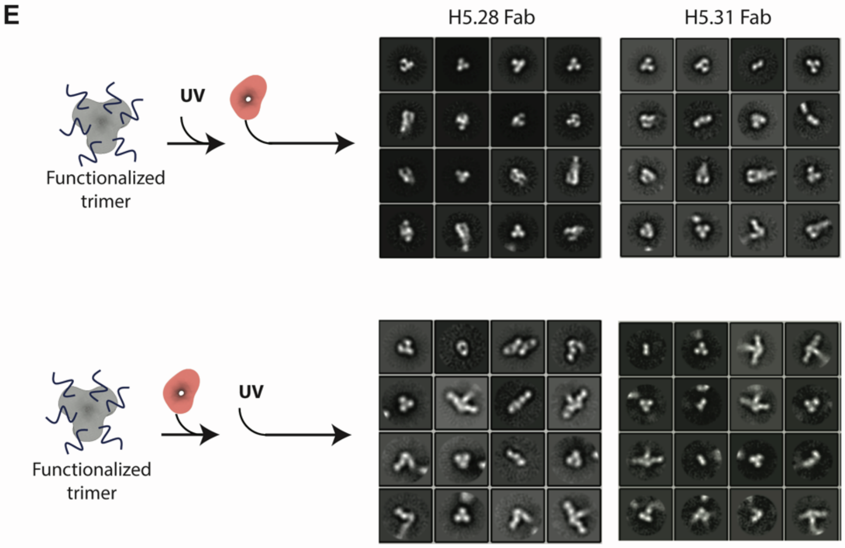

Figure S3. Biochemical and biophysical properties of the low-affinity and low-abundant Fabs. Related to Figure 3. (**A**) sample 2D class averages used to determine stoichiometry for each 3bnc117 Fab molar excess. Fab occupancy examples are highlighted. (**B**) HIV trimer occupancy distribution over a range of molar excess of 3bnc117 Fab. (**C**) Assessing affinity of the low affinity RM19D Fab by BLI. (**D**) 2D class averages of HIV trimer bound to 3bnc117 Fab and CH103 Fab (left panels). A mixture of 3bnc117 and CH103 Fabs in complex with HIV trimer in a ratio of 97:3 (3bnc117:CH103) with and without using photo-cross-linker (CL) (right panels). (**E**) 2D class averages of intermediate states of the HA protein upon H5.28 and H5.31 Fab binding.

Above, functionalization of Sulfo-SDAD to HA followed immediately by UV irradiation causes the trimer to be in a closed confirmation, with minimal antibody binding. Below, the immediate addition of two monoclonal antibodies to functionalized HA, followed UV irradiation after, allows for three antibody-antigen binding states to be visualized: 1) trimer alone, 2) HA as a dimer with two monoclonal antibodies bound and 3), HA as a monomer with a single antibody bound.

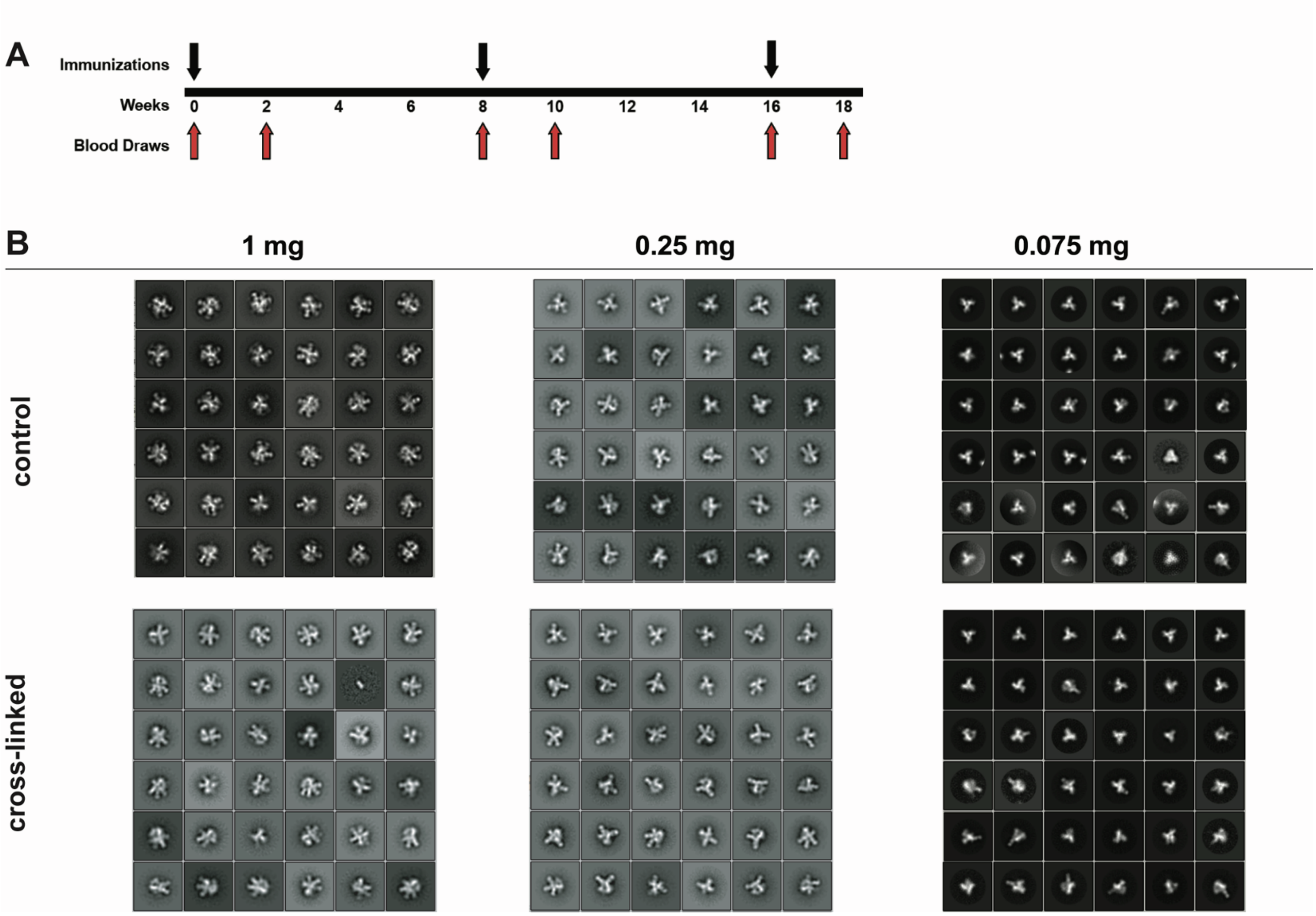

Figure S4. Immunization experiments with sera from rabbits vaccinated with the HIV immunogen.

Related to Figure 4. (**A**) Immunization schedule. Red arrows represent the weeks that blood was drawn and black arrows when rabbits were immunized with the HIV trimer. (**B**) Representative 2D class averages from the NS-EM datasets used for the generation of the composite figures presented in Figure 4.

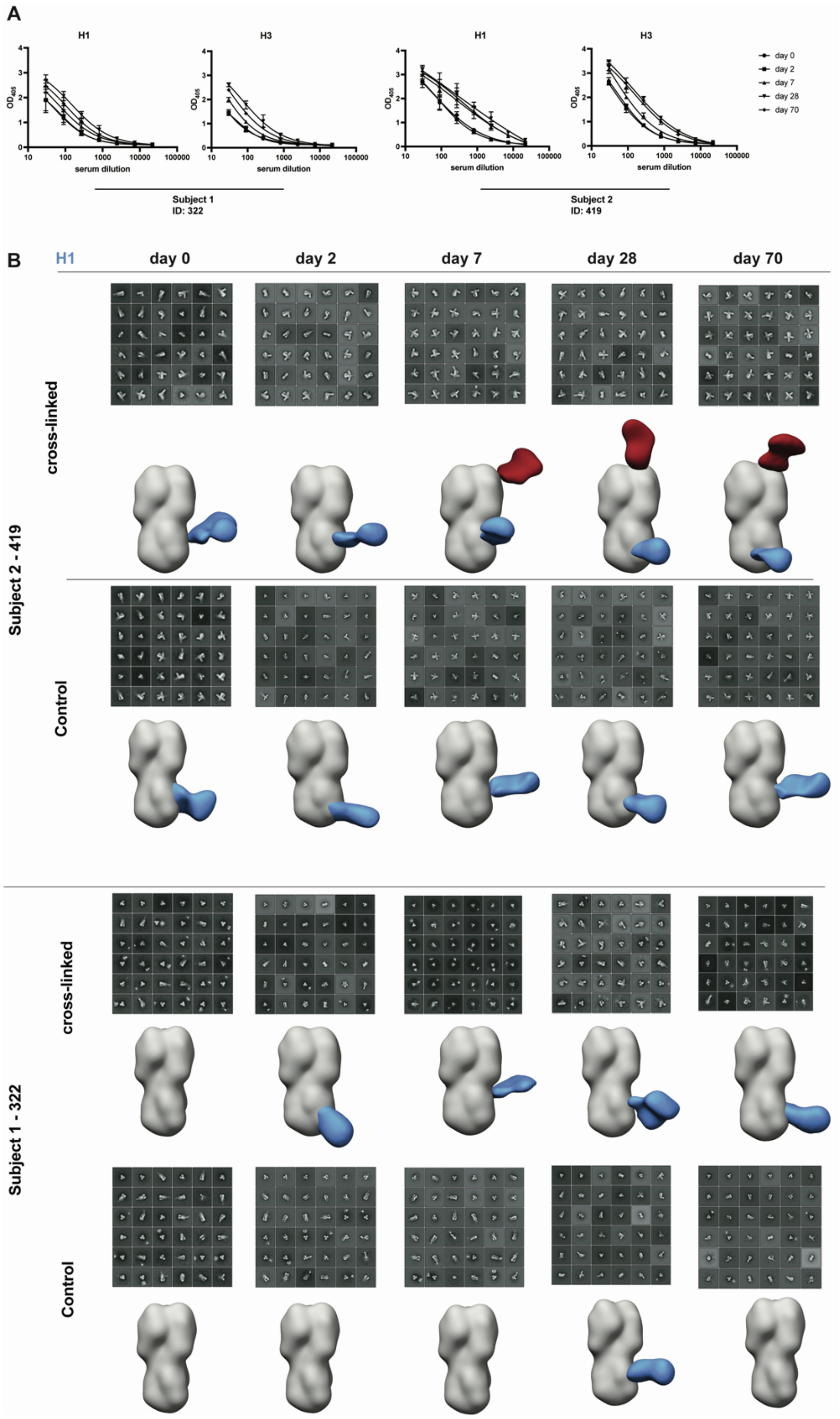

Figure S5. Extended ELISA binding titers and negative stain EMPEM analysis for HA after vaccination. Related to Figure 4. **(A)** Anti-HA binding antibody titers (EC_{50}) determined by ELISA for sera samples at different timepoints **(B-C)** Representative 2D class averages and composite models from the ns-EMPEM datasets used for the generation of the longitudinal dot plots presented in Figure 5.

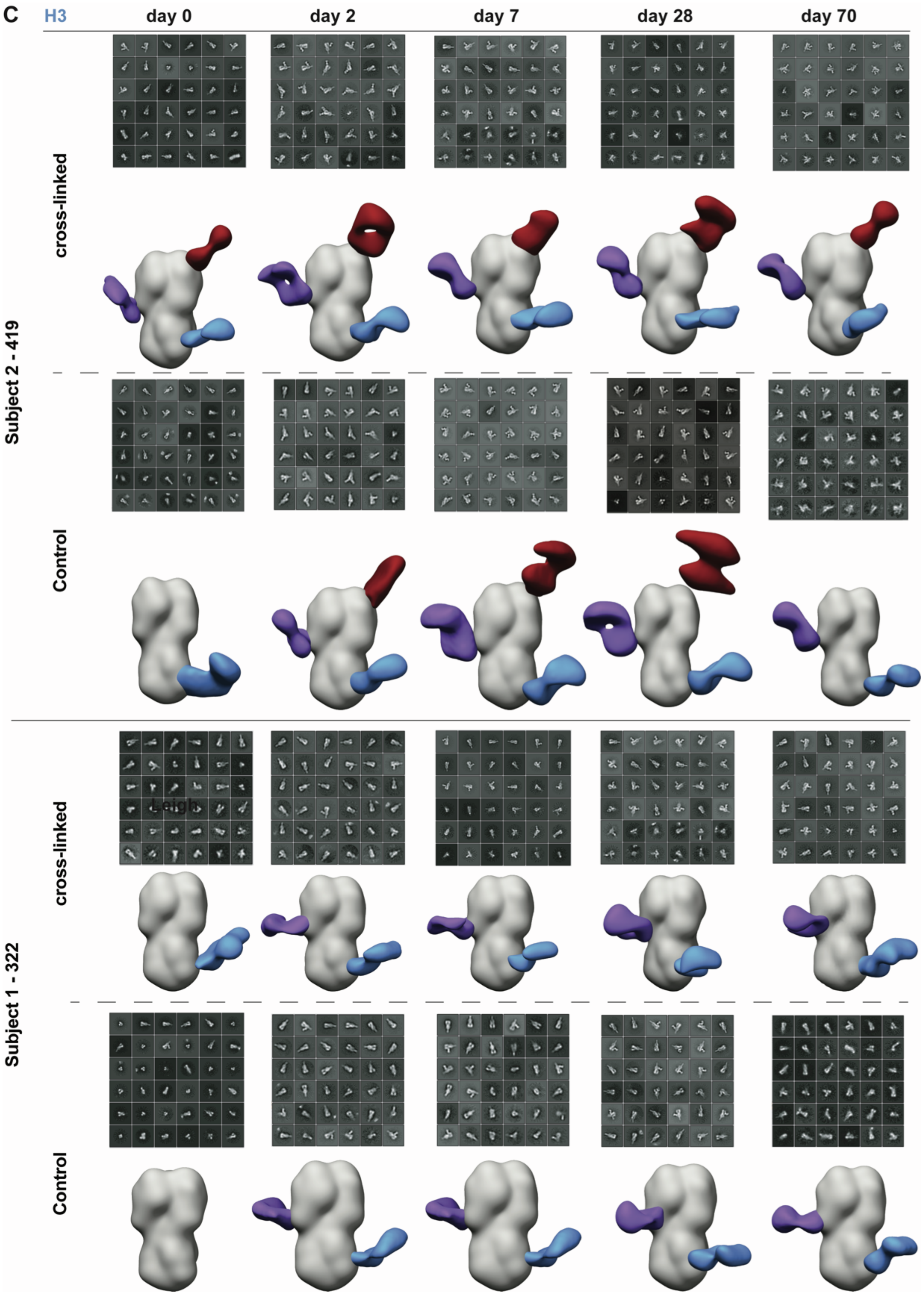

Figure S5. Extended ELISA binding titers and negative stain EMPEM analysis for HA after vaccination. Related to Figure 4. **(A)** Anti-HA binding antibody titers (EC_{50}) determined by ELISA for sera samples at different timepoints **(B-C)** Representative 2D class averages and composite models from the ns-EMPEM datasets used for the generation of the longitudinal dot plots presented in Figure 5.

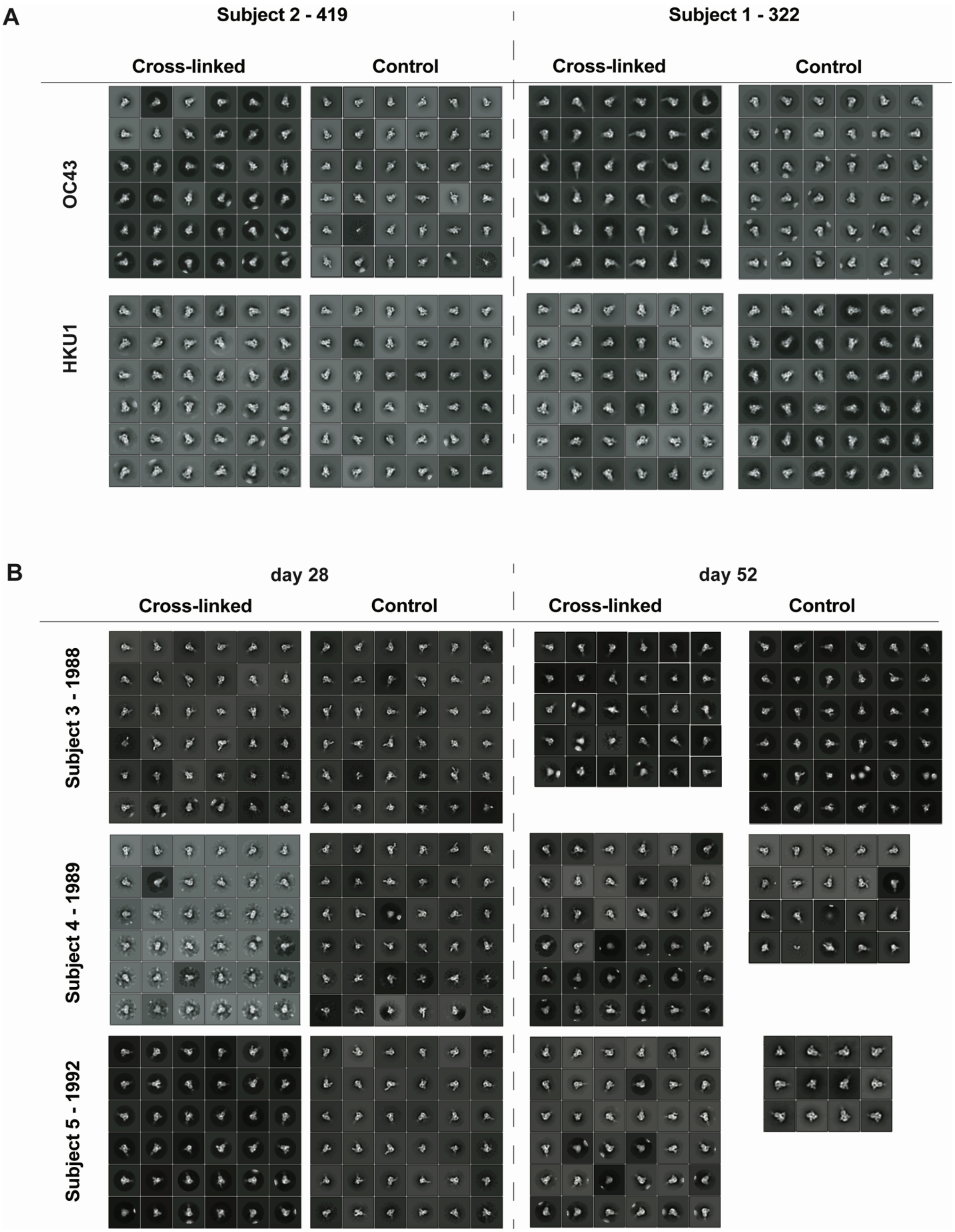

Figure S6. Extended ns-EMPEM for seasonal and pandemic HCoV during or after infection. Related to Figure 5. Representative 2D class averages from the negative stain EMPEM datasets used for the generation of composite figures of OC43, HKU1 (A) and SARS-2 (B) that are presented in Figure 5.
